# Exploring the complex relationships between coping strategies, locus of control and self-esteem with psychopathology: structural equation modeling with a special focus on clinical high-risk of psychosis

**DOI:** 10.1192/j.eurpsy.2023.2457

**Published:** 2023-10-18

**Authors:** Giulia Rinaldi, Naweed Osman, Michael Kaess, Benno G. Schimmelmann, Jochen Kindler, Frauke Schultze-Lutter, Chantal Michel

**Affiliations:** 1 University Hospital of Child and Adolescent Psychiatry and Psychotherapy, University of Bern, Bern, Switzerland; 2 Department of Psychiatry and Psychotherapy, Medical Faculty, Heinrich-Heine-University, Düsseldorf, Germany; 3 Department of Child and Adolescent Psychiatry, Centre for Psychosocial Medicine, University Hospital Heidelberg, Heidelberg, Germany; 4 University Hospital of Child and Adolescent Psychiatry, University Hospital Hamburg-Eppendorf, Hamburg, Germany; 5 Department of Psychology, Faculty of Psychology, Airlangga University, Surabaya, Indonesia

**Keywords:** clinical high risk for psychosis, competence beliefs, coping, locus of control, mental health promotion

## Abstract

**Background:**

Coping strategies, competence, and locus of control (LOC) beliefs are important predictors of mental health (MH). However, research into their complex interactions has produced mixed results. Our study investigated them further in the previously unexplored context of clinical high-risk (CHR) of psychosis.

**Methods:**

We tested six alternative structural equation models in a community sample (*N* = 523), hypothesizing a mediating role of coping and treating CHR symptoms as (i) an additional mediator or (ii) a specific outcome. Our measurement model included two latent factors of MH: (1) psychopathology (PP), consisting of presence of mental disorders, global and psychosocial functioning, and (2) self-rated health (SRH) status.

**Results:**

In the model with the best Akaike Information Criterion and the latent factors as outcome variables, maladaptive coping completely mediated the impact of maladaptive LOC on PP and SRH. Additionally, CHR symptoms partially mediated the effect of maladaptive coping on PP and SRH in the community sample, as long as sex was not entered into the model. In the clinical sample (*N* = 371), the model did not support a mediation by CHR symptoms, despite significant pathways with both coping and MH outcomes; further, competence beliefs directly impacted SRH.

**Conclusions:**

Coping strategies are an important intervention target for MH promotion, especially in the community. In clinical populations, interventions focusing on coping strategies may improve CHR symptoms, thus potentially supporting better MH, especially SRH. Additionally, due to their mostly cascading effects on MH, improving competence and LOC beliefs may also promote psychological well-being.

## 1. Introduction

Psychotic disorders are among the most frequent causes of disability-adjusted life years in adults [1] and adolescents [2] and rate second in resulting costs [3]. Psychotic episodes are mostly preceded by a prodromal phase, in which the onset of clinical high-risk (CHR) symptoms, other mental health (MH) problems, and deficits in psychosocial functioning often leads to help-seeking [4–6]. Longer duration of an inadequately treated prodromal phase is associated with negative outcomes of first-episode psychosis (FEP) [2, 7–9]. Therefore, this phase offers a unique point of intervention for an indicated prevention, aimed at reducing CHR symptoms and distress, thereby postponing or preventing manifest psychosis [10].

Despite direct associations of CHR symptoms with distress and an increased risk for psychosis [10–13], relative declines in transition rates and high rates of onset and persistence of non-psychotic disorders in CHR populations have been observed [11, 14–16]. This has generated debate regarding diagnostic specificity of CHR in predicting psychosis, with suggestions that it might be pluripotential, indicating risk for developing a range of different psychiatric conditions [17, 18]. Consequently, it was proposed that the CHR state be redefined as a transdiagnostic at-risk mental state (e.g., Clinical At-Risk Mental State; CHARMS [19]), allowing for the identification of early signs of multiple severe mental disorders. However, other studies [20–23] support the diagnostic specificity of CHR symptoms, indicating that only emergent psychotic disorders significantly differentiate between CHR patients and non-CHR help-seeking controls [21], and that the onset and persistence of non-psychotic disorders occur at a similar frequency in both groups, suggesting that a CHR status does not specifically represent a risk factor for non-psychotic disorders [21, 22].

Therefore, while the question of the diagnostic specificity of CHR status remains open, the clinical significance of CHR – for example, psychological burden, independent of conversion to a full-blown mental disorder, and negative impact on functioning – is undisputed [10–12, 19, 20, 23], and the inclusion of Attenuated Psychosis Syndrome in Section III of DSM-5 supports its diagnostic and psychopathological relevance [24], highlighting the need to focus on offering CHR patients effective interventions. Moreover, irrespective of the debate regarding pluripotentiality of the CHR state, evidence indicates some transdiagnostic relevance of the CHR state (or symptoms) in terms of (at least) comorbidity with other psychiatric disorders and syndromes [25–27]. This is reflected in new broader transdiagnostic and dimensional psychiatric taxonomies wherein efforts are currently being made to determine the most appropriate way to map CHR for psychosis into these models [28].

Relatedly, other relevant intervention targets for this population include transdiagnostic factors of core beliefs – consisting of locus of control (LOC) and competence beliefs – and coping, demonstrating dysfunctional patterns in CHR [29], FEP [29, 30], and schizophrenia patients alike [31, 32], and are regarded as possible predictors of psychosis [29]. That is, the hypothesis that typical psychotic symptoms, for example, delusions and hallucinations, result from the use of dysfunctional coping and core beliefs in response to basic symptoms (BS; self-experienced subclinical disturbances in thinking, speech, and perception) [33] and stressful stimuli [34].

Beyond their role in CHR, coping and core beliefs are also relevant for general MH quality [35–37], as reflected by multiple outcomes, including psychopathology, psychosocial functioning, and self-assessment of one’s own health status [38]. Coping is an especially important predictor of MH quality [35, 39, 40], particularly regarding stress [36] and representing either a risk (maladaptive coping, including avoidant and emotion-oriented strategies [41–43]) or protective factor (adaptive coping, including problem-focused and active strategies [44, 45]). LOC is another predictor for MH [31, 46]: internal LOC (attributing positive events to internal causes and negative ones to external factors such as chance or others) is linked to better MH outcomes and greater resilience [47], while external LOC (the opposite tendency) is associated with psychiatric disorders, including depression and schizophrenia as well as generally poorer functioning [31, 46, 47]. Thus, they can be conceptualized as adaptive and maladaptive, respectively. Finally, competence beliefs, including self-efficacy and self-esteem [48, 49], are strongly associated with MH quality [37, 50], with higher competence beliefs being related to better psychosocial functioning [37, 51].

Investigations into the interactions between coping, core beliefs, and MH, involving mainly community samples but also including a minority of clinical samples, have led to contradictory findings in both populations, indicating a mediating role of coping [52–54] or of core beliefs [49, 55, 56]. A recent meta-analysis [36] – also mostly, but not exclusively, using community samples – supported a mediation by coping on the influence of core beliefs on MH. Specifically, maladaptive coping mediated the relationship between maladaptive LOC and MH problems. Moreover, both adaptive and maladaptive LOC showed a direct influence on MH problems, independent of coping.

In the present study, we extended the meta-analytical and mediation model [36] that had mixed community and clinical samples by first exploring alternative structural equation models (SEM) in a community sample and then examining their validity in a clinical sample. In addition to general psychopathology, we focused on CHR symptoms, in virtue of their association with MH quality [10–12] as well as coping and core beliefs [29]. The aims of the present study were:

1. To explore the association between core beliefs and MH outcomes, in both a community and a clinical sample, assuming a mediation by coping. Specifically, based on the metanalytical model [36], we anticipated that the effect of competence beliefs and adaptive LOC on MH outcomes would be mediated by adaptive coping, and that the effect of maladaptive LOC would be mediated by maladaptive coping.
2. To investigate the specific placement of CHR symptoms in these interactions.
  Based on the metanalytical model [36], we did not expect relationships between competence beliefs and adaptive LOC, and maladaptive coping or between maladaptive LOC and adaptive coping, and therefore we did not include these relationships in the models.

## 2. Methods

### 2.1. Participants and recruitment procedure

Cross-sectional data from a community and a clinical sample were used in the current study. The former comprised 523 participants in the first follow-up assessment of the Bern Epidemiological At-Risk (BEAR) study [57, 58], whose core beliefs and coping strategies were evaluated in an add-on study (Supplementary eFigure 1, Supplementary eText 1). Inclusion criteria were absence of a psychotic disorder and fluency in German.

The second sample included 378 patients of the Bern Early Recognition and Intervention Centre for mental crisis (FETZ Bern), assessed between November 2009 and July 2022. Inclusion criteria were informed consent to the use of collected data for scientific purposes, age above 13 years (to allow for the assessment of all BS), and sufficient German-language skills. For more information regarding recruitment and assessment procedures in the BEAR study [57] or FETZ Bern [59], see Supplementary eTexts 1–4.

### 2.2. Assessments

#### 2.2.1. Mental disorders

The Mini-International Neuropsychiatric Interview (MINI) [60] was used to assess current presence of Axis-I mental disorders according to the *Diagnostic and Statistical Manual of Mental Disorders*, Fourth Edition (DSM-IV) [61]. The presence of each disorder was indicated by a score of 1 in the corresponding scale; their sum score (0–36) was used in analyses.

#### 2.2.2. CHR symptoms

Two approaches are used for the assessment of CHR states: (i) ultra-high-risk (UHR) criteria and (ii) BS criteria (Supplementary eTable 1). The Structured Interview for Psychosis Risk Syndromes (SIPS) [62] was used to assess the presence of UHR symptoms (attenuated (APS) or brief intermittent psychotic symptoms (BIPS)). For each of the positive items (P1–P5; Supplementary eTable1), participants received a score of 1 if they presented symptoms rated between 3 and 5 (APS) or equal to 6 (BIPS), irrespective of whether or not the APS/BIPS in question met requirements for onset/worsening and frequency of the UHR criteria that are very infrequent in the general population [57, 62]. Scores were then added in a sum score (0–5).

The presence of the BS criteria, cognitive disturbances (COGDIS), and cognitive-perceptive basic symptoms (COPER) was assessed with the Schizophrenia Proneness Instrument–Adult [63] and Child and Youth [64] versions. Irrespective of the frequency and novelty requirements for BS criteria that are also infrequent in the community [33], the presence of each criterion-relevant BS (Supplementary eTable1) was indicated by a score of 1, and a sum score (0–14) was obtained.

#### 2.2.3. Self-rated health

Self-rated health was evaluated via the EuroQoL-5D, three-level version (EQ-5D-3L) [65], assessing three degrees of severity across five dimensions of health, from which we obtained a sum score (0–100) [66, 67]. Participants’ self-rating of their current health state on the EQ-5D-3L analog scale (0–100, “worst” to “best imaginable health state”) was also included in our models.

#### 2.2.4. Global, social, and occupational functioning

Functioning was assessed with both the Global Assessment of Functioning (GAF) scale, in which psychiatric symptoms are considered, and the Social and Occupational Functioning Assessment Scale (SOFAS) for the evaluation of functioning independently from symptoms [61].

#### 2.2.5. Core beliefs

The German Competence and Control Beliefs Questionnaire (FKK) [68] was used to evaluate these constructs by means of Self-Concept (FKK-SK; 8 items), Internality (FKK-I; 8 items), and Externality (FKK-PC; 16 items) scales. These were conceptualized in our models as competence beliefs (FKK-SK; as recommended in [68], see also [69]), adaptive (FKK-I), and maladaptive LOC (FKK-PC; “internality” and “externality” are synonyms for internal, that is, adaptive, and external, that is, maladaptive, LOC, respectively [31, 70]). Analyses were conducted with the normative T-values of each scale’s sum score, obtained from ratings in their respective items on a bipolar six-level scale.

#### 2.2.6. Coping strategies

Positive and negative coping was assessed via the German Stress Coping Questionnaire, adult (SVF-120) [71] and children/adolescents (SVF-KJ) [72] versions. In each item, the frequency of use of different coping strategies can be rated on a 0–4 Likert scale (“not at all”–“in any case”). In our analyses, we used the relative normative T-values to the sum scores of the global scales Positive and Negative Coping Strategies to represent adaptive and maladaptive coping, respectively.

#### 2.2.7. Sociodemographic variables

Age, level of education, and sex were included in the models as possible confounding variables, the latter only at a later stage during a sensitivity analysis.

Further details regarding instruments can be found in Supplementary eText 5.

### 2.3. Statistical analyses

Data analyses were performed in RStudio, version 4.1.1, using the lavaan package for preliminary exploratory and confirmatory factor analyses (EFA, CFA) and testing alternative SEMs, and the sempower package for power analysis. The community sample served as the model generation; the clinical sample as model validation sample.

First, an EFA was conducted using variables pertaining to participants’ MH (presence of Axis-I mental disorders and self-rated health) based on Spearman correlation matrices and using Oblimin rotation, allowing intercorrelation of factors. Pairwise deletion was applied, excluding one participant who was missing 20% of the data. Based on EFA results, we proceeded with a two-factor CFA.

Finally, six alternative SEMs were computed using the maximum likelihood estimator [73]. After a pairwise deletion of five observations with missing data, the analysis was conducted on 518 participants from the community sample. Along with the EFA/CFA factors, variables included age, education, standard T-values for competence beliefs (FKK-SK), maladaptive LOC (FKK-PC), adaptive LOC (FKK-I), adaptive and maladaptive coping, presence of BS and APS/BIPS, or alternatively presence of either of CHR symptoms. A Tucker-Lewis index (TLI) ≥0.90, a comparative fit index (CFI) ≥0.95, a standardized root mean square residual (SRMR) ≤0.08, a root mean square error of approximation (RMSEA) ≤0.06, as well as a 90% confidence interval (CI) not containing 0.08 indicate excellent model fit [74]. As the Chi-squared test is sensitive to sample size and often results in model rejection when working with large samples [75], we focused on the aforementioned indices in evaluating model fit. After comparing the models’ Akaike Information Criterion (AIC) [76] and Bayesian Information Criterion (BIC) [77], one model was selected as fitting the data best; this was validated in the clinical sample.

The clinical sample (N = 371) presented higher amounts of missing data (9.58%). After applying listwise deletion to 51 participants missing >50% [78] of their data, we used a multiple imputation method on data missing from the remaining 327 subjects [79].

To control for sex differences, we conducted a sensitivity analysis by including sex in the chosen model and testing it again in both samples. Here the introduction of a categorical variable in the model required the use of the Weighted least squares and variance-adjusted estimator [73]. We chose this procedure instead of directly including sex in the six alternative SEMs because using this estimator would not have allowed a statistically valid selection of one best-fitting model. Finally, in all samples, we tested all possible mediation pathways indicated in the selected model for significance and calculated their respective 95% bias-corrected bootstrap CIs.

## 3. Results

### 3.1. Sample characteristics

The two samples differed in sex (more males in the clinical sample), age, and highest educational level (both lower in the clinical sample), as well as in clinical and functional variables, with lower functioning and more severe psychopathology in the clinical sample (Table 1).

**Table 1.** Sample characteristics and group comparison

		Community sample (*N* = 523)		Clinical sample (*N* = 371)^a^		
		*n*	%	*n*	%	Statistics; effect size
Age (mean ± SD, median, range)		33.4 ± 7.8, 35.00, 19.00–45.00		18.94 ± 4.51, 17.44, 12.98–40.30		*U* = 186,426, *p* < 0.001; *r* = 0.757
Sex (male)		204	39.0	179	47.4	χ^2^ = 15.956, *p* < 0.001; *V* = 11.166
Highest professional education (ISCED level)						*U* = 142,062, *p* < 0.001; *r* = 0.456
	Early childhood education (ISCED 0)	0	0	4	1.1	
	Primary school or school for special needs (ISCED 1)	0	0	6	1.6	
	Secondary school (ISCED 2)	5	1.0	108	28.6	
	Highschool (ISCED 3.4)	8	1.5	10	2.6	
	Highschool-level professional education (ISCED 3.5)	36	6.9	38	11.9	
	Post-secondary non-tertiary education (ISCED 4)	6	1.1	1	0.3	
	Short-cycle tertiary education (ISCED 5)	256	48.9	141	37.3	
	Master (ISCED 7)	205	39.2	45	11.9	
	Doctoral (ISCED 8)	7	1.3	0	0	
SOFAS score (mean ± SD, median, range)		84.80 ± 6.66, 88, 40.00–100.00		59.35 ± 12.97, 60, 30.00–95.00		*U* = 174,438, *p* < 0.001; *r* = 0.775
GAF score (mean ± SD, median, range)		81.70 ± 9.84, 87.0, 36.00–95.00		51.86 ± 12.51, 53, 21.00–90.00		*U* = 176,177, *p* < 0.001; *r* = 0.770
Current Axis-I disorders, sum score (mean ± SD, median, range)		0.21 ± 0.61, 0, 0.00–6.00		1.06 ± 1.06, 1, 0.00–6.00		*U* = 37,924, *p* < 0.001; *r* = 0.483
Current CHR symptoms, sum score (mean ± SD, median, range)		0.44 ± 0.61, 0, 0.00–5.00		4.28 ± 3.29, 3, 0.00–14.00		*U* = 17,212, *p* < 0.001; *r* = 0.698
Current UHR symptoms, sum score (mean ± SD, median, range)		0.15 ± 0.43, 0, 0.00–3.00		1.74 ± 1.25, 2, 0.00–5.00		*U* = 25,606, *p* < 0.001; *r* = 0.687
Current basic symptoms, sum score (mean ± SD, median, range)		0.29 ± 0.60, 0, 0.00–4.00		2.63 ± 2.51, 2, 0.00–10.00		*U* = 28,810, *p* < 0.001; *r* = 0.608

Abbreviations: CHR, clinical high risk; *r*, Pearson’s r; SOFAS, Social and Occupational Functioning Assessment Scale; UHR, ultra high risk; *U*, Mann-Whitney U test, *V*, Cramer’s V; χ^2^, Chi-squared.

a In the FETZ sample, 18 participants (4.8%) were missing data about their education level (ISCED), 30 participants (7.9%) were missing data about their SOFAS score, 26 participants (6.9%) were missing data about their GAF score, 85 participants (6.9%) were missing data about their current Axis-I disorders, 46 participants (12.2%) were missing data about their current CHR symptoms, 26 participants (6.9%) were missing data about their current UHR symptoms, 45 participants (11.9%) were missing data about their current basic symptoms.

### 3.2. EFA and CFA in the community sample

Results of the EFA (Supplementary eTable 2) indicate two correlated latent factors (factor correlation 0.34): (i) psychopathology (PP) and (ii) self-rated health (SRH). The model’s fit to the community sample data was excellent overall (RMSR = 0.01, TLI = 0.98, RMSEA = 0.059). The CFA (N = 522) confirmed the two-factor structure (Supplementary eTable 3), showing very good model fit (CFI = 0.996, TLI = 0.990, RMSEA = 0.062, SRMR = 0.032).

### 3.3. SEM models in the community sample

The resulting latent factors were included in six alternative SEM models (Supplementary eText 6). In all models, positive and negative coping strategies mediated the effect of competence beliefs and adaptive and maladaptive LOC on the latent MH factors PP and SRH.

**Figure 1.**
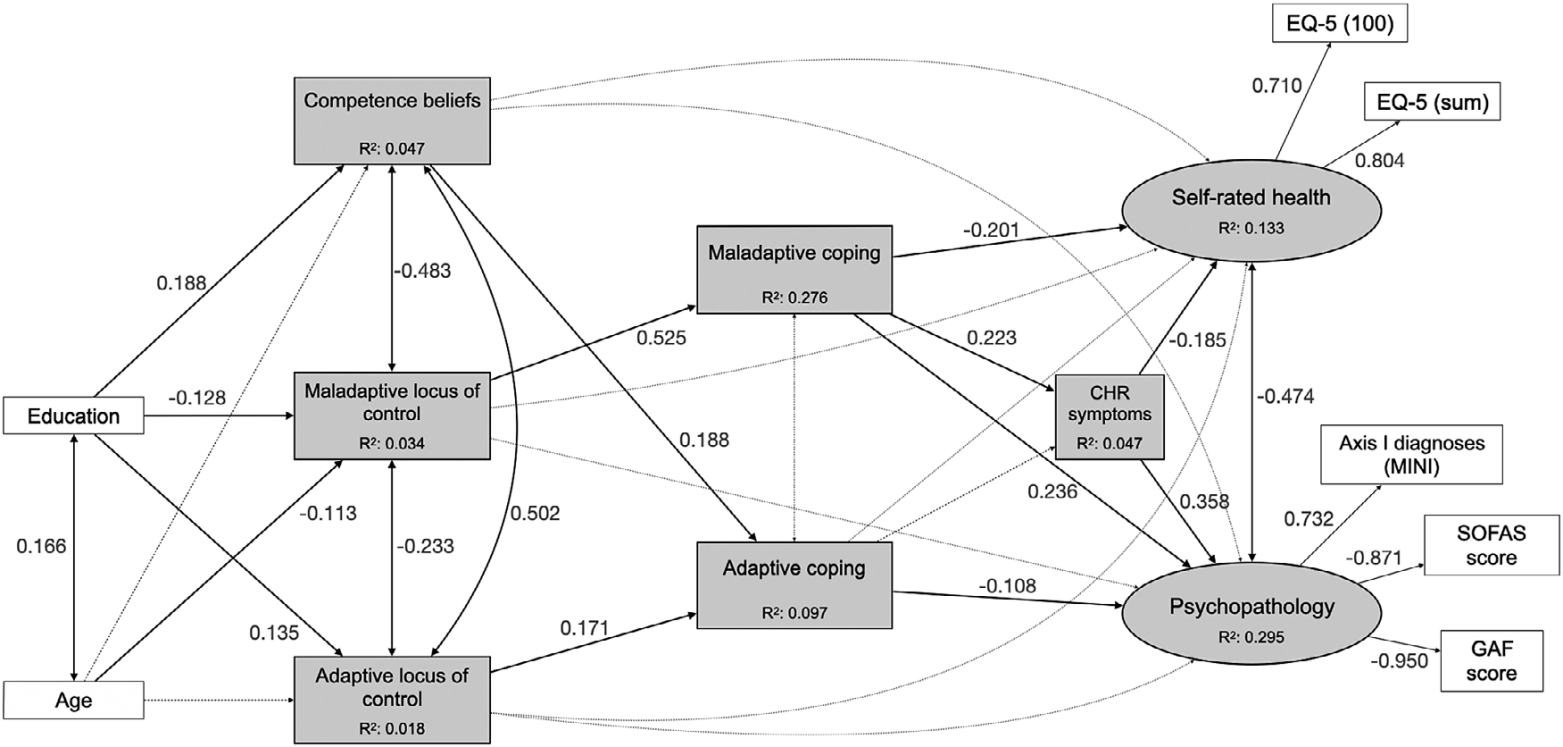
Model 1.2 in the community sample. Rectangles represent observed variables; ovals represent unobserved latent variables; black lines with double-ended arrows represent covariances; black lines with single-ended arrows represent significant paths; dashed gray lines with double- or single-ended arrows represent non-significant covariances or regression paths, respectively; numbers next to the lines indicate coefficients of significant standardized regressions and covariances, or factor loadings; the coefficients of non-significant covariances and regressions are not reported here to facilitate the figure’s interpretation; see Table 2 and Supplementary eTable 5 for further details. CHR: clinical high risk; EQ-5 (100): score on the 0–100 analog scale of the EuroQoL-5D, three-level version (EQ-5D-3L); EQ-5 (sum): sum score on EQ-5D-3L – see Supplementary eText 5 for details; GAF, Global Assessment of Functioning; MINI, Mini-International Neuropsychiatric Interview; SOFAS, Social and Occupational Functioning Scale.

Fit indices and power ranged from acceptable to excellent, except for TLI, which was equally poor for all models (Supplementary eTable 4). Comparison of their AIC and BIC indices, with emphasis on AIC, indicated model 1.2 (Figure 1, Table 2, Supplementary eTable 5) as best fitting the BEAR data (CFI = 0.923, TLI = 0.863, RMSEA = 0.086, 90% CIs = 0.075, 0.098, SRMR = 0.055, power >0.999, AIC = 39,484.669, BIC = 39,684.418), although model 3.2, with CHR symptoms as an outcome of SHR and PP, had lower BIC (BIC = 39,677.074, AIC = 39,485.825). Though the two models had a similarly good fit to the data, AIC was emphasized in model selection, being more relevant to our testing of a complex system of interactions with unknown underlying structure [80], and since BIC can lead to underfitting when working with large samples, non-nested models, and data not following a multivariate normal distribution [81].

**Table 2.** Standardized regression coefficients (β) and *p* values for relevant paths in model 1.2

	Community sample (*N* = 518)		Clinical sample (*N* = 327)	
	β	p	β	p
**Psychopathology** **(PP)**				
Maladaptive coping	0.236	<0.001**	*−0.053*	*0.401*
Adaptive coping	−0.108	0.009*	*−0.080*	*0.212*
CHR symptoms	0.358	<0.001**	0.313	<0.001**
**Maladaptive coping**				
Maladaptive LOC	0.525	<0.001**	0.433	<0.001**
**Adaptive coping**				
Competence beliefs	0.188	<0.001**	0.275	<0.001**
Adaptive LOC	0.171	<0.001**	0.266	<0.001**
**Self** **-rated** **health** **(SRH)**				
Maladaptive coping	−0.201	0.001**	*−0.007*	*0.927*
CHR symptoms	−0.185	<0.001**	−0.434	<0.001**
Competence beliefs	*−0.030*	*0.636*	0.230	0.004*
**CHR symptoms**				
Adaptive coping	*−0.003*	*0.947*	−0.153	0.005*
Maladaptive coping	0.223	<0.001**	0.204	<0.001**
**Competence beliefs**				
ISCED level	0.188	<0.001**	*0.101*	*0.113*
**Adaptive LOC**				
ISCED level	0.135	0.002*	*−0.020*	*0.756*
**Maladaptive LOC**				
ISCED level	−0.128	0.004*	*−0.092*	*0.150*
Age	−0.133	0.010*	*0.063*	*0.323*

*Note*: *italics*, not significant, significant in the other sample.

** *p* < 0.001;

* *p* < 0.05.

In the community sample, maladaptive coping completely mediated the effect of maladaptive LOC on PP, SRH, and CHR symptoms (Table 3), and adaptive coping mediated the impact of competence beliefs, but not of adaptive LOC, on PP. Additionally, CHR symptoms partially mediated the effect of maladaptive coping on PP and SRH. No significant direct effects of competence beliefs and LOC on PP or SRH were detected.

**Table 3.** Mediation effect analyses, 95% bias-corrected bootstrap confidence intervals

	Community sample (*N* = 518)			Clinical sample (*N* = 327)		
Mediation pathway	Standardized coefficient	*p*	95% CI	Standardized coefficient	*p*	95% CI
*Competence beliefs–adaptive coping–PP*						
Indirect effect	−0.020	0.040*	*−0.002,* *0.000*			
Total effect	*−0.053*	*0.403*	*−0.009, 0.003*			
*Competence beliefs–adaptive coping–CHR symptoms*						
Indirect effect				*−0.028*	*0.124*	*−0.024, 0.001*
Total effect				−0.224	0.002*	−0.131, −0.030
*Adaptive LOC–adaptive coping–CHR symptoms*						
Indirect effect				*−0.027*	*0.107*	*−0.022, 0.001*
Total effect				*0.015*	*0.805*	*−0.037, 0.046*
*Adaptive LOC–adaptive coping–PP*						
Indirect effect	*−0.018*	*0.071*	*−0.002,* *0.000*			
Total effect	*−0.060*	*0.264*	*−0.008, 0.002*			
*Maladaptive LOC–maladaptive coping–SRH*						
Indirect effect	−0.106	0.026*	−0.200, −0.019			
Total effect	−0.181	0.011*	−0.339, −0.064			
*Maladaptive LOC–maladaptive coping–PP*						
Indirect effect	0.124	0.003*	0.003, 0.011			
Total effect	0.205	0.001**	0.005, 0.017			
*Maladaptive LOC–maladaptive coping–CHR symptoms*						
Indirect effect	0.111	<0.001**	0.005, 0.016	*0.027*	*0.302*	*−0.007, 0.030*
Total effect	0.133	0.003*	0.005, 0.020	0.155	0.009*	0.014, 0.097
*Maladaptive coping–CHR symptoms–SRH*						
Indirect effect	−0.039	0.047*	−0.090, −0.011	*−0.026*	*0.304*	*−0.108, 0.022*
Total effect	−0.240	0.008*	−0.404, −0.061	*−0.033*	*0.704*	*−0.242, 0.162*
*Maladaptive coping–CHR symptoms–PP*						
Indirect effect	0.076	0.004*	0.001, 0.007	*0.019*	*0.322*	*−0.001, 0.003*
Total effect	0.312	<0.001**	0.008, 0.024	*−0.034*	*0.607*	*−0.007, 0.005*
*Adaptive coping–CHR symptoms–SRH*						
Indirect effect				*0.043*	*0.101*	*−0.004, 0.125*
Total effect				*0.046*	*0.577*	*−0.131, 0.257*
*Adaptive coping–CHR symptoms–PP*						
Indirect effect				*−0.031*	*0.101*	*−0.003, 0.000*
Total effect				*−0.110*	*0.102*	*−0.012, 0.000*

*Note*: *italics*, not significant; value missing, indirect effect was not analyzed in the corresponding sample.

** *p* < 0.001;

* *p* < 0.05.

In the sensitivity analysis, introducing sex as an exogenous variable in model 1.2 (Supplementary eFigure 8, Supplementary eTable 6) fit to the community sample data and power were excellent across all indices (CFI = 0.989, TLI = 0.982, RMSEA = 0.04, 90% CIs = 0.027, 0.045, SRMR = 0.045, power >0.999). Direct paths between the variables remained unaltered, but all mediation effects were insignificant. Competence beliefs newly showed a direct effect on PP.

### 3.4. SEM model 1.2 in the clinical sample

**Figure 2.**
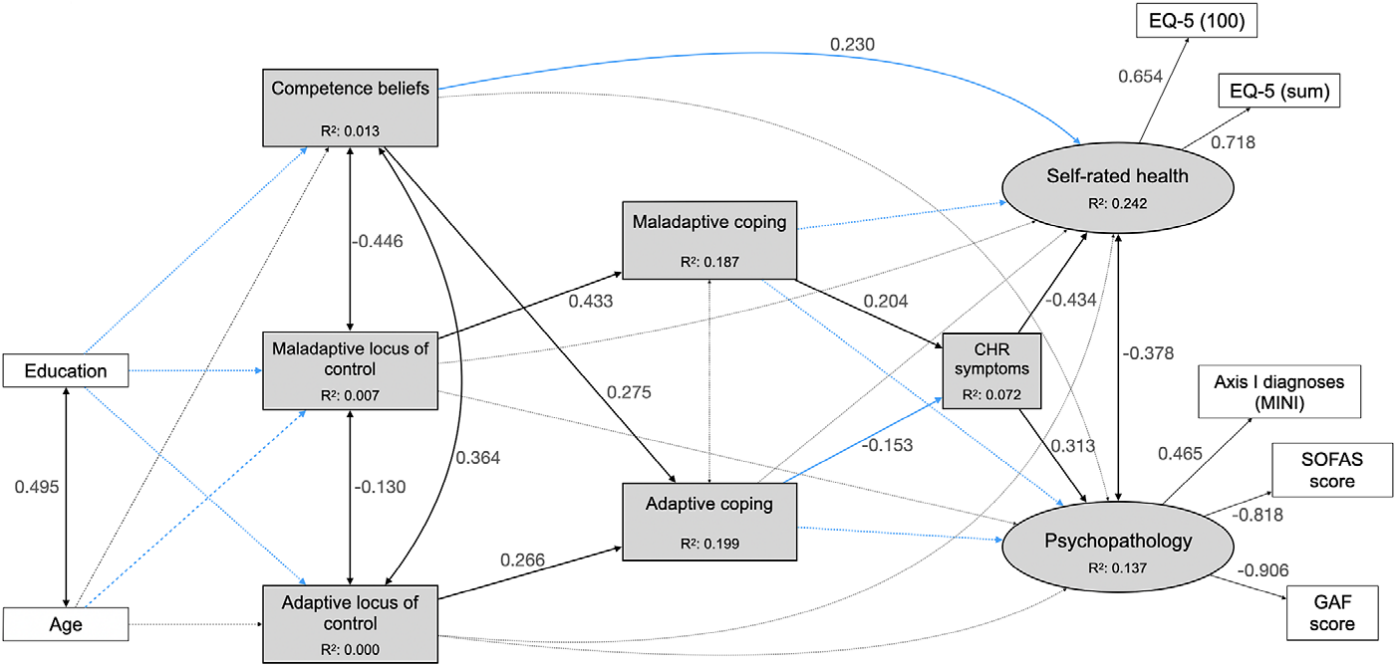
Model 1.2 in the clinical sample. Rectangles represent observed variables; ovals represent unobserved latent variables; black lines with double-ended arrows represent covariances; black lines with single-ended arrows represent significant paths; gray lines with double- or single-ended dashed arrows represent non-significant covariances or regression paths, respectively; numbers next to the lines indicate coefficients of significant standardized regressions and covariances, or factor loadings; the coefficients of non-significant covariances and regressions are not reported here to facilitate the figure’s interpretation; see Table 2 and Supplementary eTable 5 for further details. Blue lines indicate differences from results of testing in the community sample. CHR: clinical high risk; EQ-5 (100): score on the 0–100 analog scale of the EuroQoL-5D, three-level version (EQ-5D-3L); EQ-5 (sum): sum score on EQ-5D-3L – see Supplementary eText 5 for details; GAF, Global Assessment of Functioning; MINI, Mini-International Neuropsychiatric Interview; SOFAS, Social and Occupational Functioning Scale.

Next, we tested model 1.2 in the clinical sample (Figure 2). Compared to the community sample, model fit decreased, with CFI (0.865) and TLI (0.761) indicating poor fit, while RMSEA (0.099, 90% CIs = 0.085, 0.114) remained acceptable and SRMR (0.073) and power (0.986) excellent (Table 2, Supplementary eTable 5).

Maladaptive and adaptive coping no longer impacted SRH or PP directly, and neither adaptive nor maladaptive LOC significantly affected the MH outcome variables. Competence beliefs, however, newly directly impacted SRH, which, compared to the community sample model, was more strongly associated with CHR symptoms. Mediation analyses (Table 3), however, revealed no significant mediation by CHR symptoms in the effect of both adaptive and maladaptive coping on SRH and PP. Furthermore, no significant mediation of coping in the relationship of competence beliefs and LOC, and CHR symptoms was found.

The sensitivity analysis (Supplementary eFigure 9, Supplementary eTable 7) led to an increase in goodness of fit and power after including sex in the model. All indices except TLI (0.898) showed values ranging from good to excellent (CFI = 0.942, RMSEA = 0.068, 90% CIs = 0.053, 0.083, SRMR = 0.068, power = 0.994).

Results did not vary except for a newly significant direct effect of competence beliefs on PP and a significant covariation between adaptive and maladaptive coping (*s* = −0.136, *p* < 0.001). No mediation effect was significant.

## 4. Discussion

### 4.1. Association between core beliefs and MH outcomes

Our first hypothesis of a mediation by coping in the association between core beliefs and MH was partially supported by findings in the community sample. Aligning with the metanalytical model mostly generated on community samples [36], maladaptive coping completely mediated the effect of maladaptive LOC on CHR symptoms, PP, and SRH, while adaptive coping only mediated the association between competence beliefs and PP. While this suggests that treating maladaptive LOC and coping may promote MH in the community, the lack of mediation effects in the sensitivity model, that is, after the inclusion of sex, calls for more research into the role of sex in these associations.

Unexpectedly, but aligning with conflicting results in the two clinical samples of the metanalytical model [36], coping did not mediate the impact of core beliefs on MH in the clinical sample. Rather, adaptive and maladaptive beliefs were associated with their coping counterparts. Coping had direct effects on CHR symptoms, which were directly associated with MH outcomes. Newly, the total effects of maladaptive LOC and competence beliefs on CHR symptoms became significant, and competence beliefs were directly linked to SRH. A possible reason is that in clinical populations, both adaptive and maladaptive coping might specifically focus on CHR symptoms, rather than overall MH quality, as our results in the community sample suggest with lower rates of CHR symptoms. Therefore, treatment targeting coping strategies in these populations might help manage and reduce CHR symptoms, preventing maladaptive coping from acting as a trigger for CHR symptoms, exacerbating them, or worsening their outcome [82]. Further, in light of our findings indicating a direct effect of competence beliefs on SRH, and of competence beliefs and LOC on coping, challenging maladaptive core beliefs may also have a positive impact on MH quality. In contrast to the metanalytical model [36], we found no direct effects of LOC on MH outcomes. Possible explanations relate to differences in our study, including added complexity of our model with three MH variables and differing conceptualizations of MH (e.g., including measures of functioning in our study).

Results indicate the need for more group-dependent research on the impact of the severity of psychopathology – and possibly type and operationalization of psychopathology – on the association and potential mediation effects of core beliefs and coping strategies with MH, as different levels of engagement with the mental healthcare system might act as an additional mediator or moderator. Such future studies will shed light on the most relevant targets for promoting MH, that is, core beliefs, coping, or both.

### 4.2. Role of CHR symptoms

To our knowledge, the present study was the first to explore CHR symptoms in the context of the interactions between core beliefs, coping, and MH, in both community and clinical samples. In the model selected as the best fit for the data, CHR symptoms were included as a contributor of MH outcome. However, the alternative model with CHR symptoms as an outcome of PP and SRH performed similarly well, indicating a strong association (albeit with unclear direction/placement) between MH variables and CHR in both samples, even after controlling for sex differences. Significant mediation effects of CHR symptoms in the relationship between coping and PP and SHR were found only in the community sample model disregarding sex but in no other model, possibly related to the cross-sectional nature of our study, preventing the drawing of definitive causal conclusions. Further factors that might help explain the differences between the community and clinical samples are (i) the differences in prevalence of CHR symptoms in the two samples, which may influence their role in relation to the other variables in our model as well as the results of our analyses; (ii) the impact of the additional burden of higher psychopathology and more severe functioning deficits in the clinical sample, which is generally more unwell compared to the community sample. Regardless, findings support some transdiagnostic relevance of CHR (regarding broader psychopathology and in relation to transdiagnostic factors) while simultaneously highlighting the challenge of accurately mapping CHR into broader psychopathological systems.

Aligning with earlier research on patients meeting UHR criteria [82, 83], maladaptive coping was more strongly and frequently significantly associated with CHR symptoms compared to adaptive coping. Whereas adaptive coping styles were stable in UHR patients, maladaptive coping more likely changed over time and was related to corresponding changes in UHR symptoms in a UHR sample [82] and, in a community sample, was bidirectionally related over time to psychotic-like experiences [84], which, however, may be a poor estimate of clinician-assessed CHR symptoms [85]. With maladaptive coping also negatively impacting functioning and likely other clinical factors such as severity of symptomatology, including depression or personality traits, interventions that challenge coping strategies – and core beliefs – might be most appropriate for populations in early stages of mental disorders or with subclinical MH problems [83].

### 4.3. Strengths and limitations

The large size of both the community and clinical samples in this study and their separate analysis provide a comprehensive view of CHR symptoms and their associations with important transdiagnostic factors related to MH and some important first insights into the potential differences between community and clinical samples. Further, the assessment of MH variables in clinical interviews conducted by highly trained psychologists, and the comprehensive definition of CHR symptoms not only by UHR but also BS, adds to data validity.

The lack of control for ongoing psychotherapeutic treatment, which might have affected several variables, may be regarded as a limitation that our study shares with most comparable studies [36]. Moreover, despite growing evidence regarding their impact on CHR outcomes, especially on psychosocial functioning [86–88], we did not include negative CHR symptoms in our models, as they were only assessed in the clinical sample and, therefore, a meaningful comparison with the community sample would not have been possible. The role of psychotherapy and negative symptoms should be explored in future research.

Additionally, for reasons of sample size and power, we opted against recommendations [89] to only impute on variables missing <5% of data but applied multiple imputation to the missing data to the SVF 120/KJ and EQ-5D-3L in the clinical sample as well, potentially constituting a statistical limitation. Furthermore, especially for the low number of participants meeting CHR criteria in the community sample (4.97%), we could not perform sensitivity analyses in CHR persons, limiting comparability with studies on CHR samples [82, 83]. Lastly, as only the model with the lowest AIC – an index that penalizes models less for free parameters and favors more saturated models compared to BIC – was further processed; other possible relevant mediations, in particular PP and SRH in model 3.2 with the lowest BIC, remained unexplored.

### 4.4. Future directions and conclusion

Our findings support evidence of community studies of a mediation role of coping in the relationship of MH variables with core beliefs, although this role might differ between sexes and may decrease with increasing MH problems. Results in the clinical sample suggest a more complex interplay of the examined variables compared to the community sample, thus indicating the need for more group-specific analyses in future studies. Considering this and the higher severity of psychopathology and functioning deficits, treatment in this population may need to be more comprehensive and tailored to target multiple factors influencing MH outcomes, including coping strategies and core beliefs, to address the specific challenges faced by help-seeking individuals. Regarding CHR symptoms, a clear association with PP and, especially, SRH became evident in all models, with inconclusive results about their constellation. Future prospective studies should further examine the transdiagnostic factors coping and core beliefs, their relationship with CHR symptoms, and their emergence of manifest mental disorders. Overall, our results contribute to existing evidence that coping strategies, competence beliefs, and LOC represent worthwhile targets for the promotion of MH and shed further light on their complex interactions.

## Supporting information

Rinaldi et al. supplementary material Rinaldi et al. supplementary material

Rinaldi et al. supplementary material Rinaldi et al. supplementary material

## Supplementary material

The supplementary material for this article can be found at http://doi.org/10.1192/j.eurpsy.2023.2457.
