# Supplementary material for: Exploring the complex relationships between coping strategies, locus of control and self-esteem with psychopathology: structural equation modeling with a special focus on clinical high-risk of psychosis
Source: Eur Psychiatry. 2023 Oct 18;66(1):e88. doi: 10.1192/j.eurpsy.2023.2457 (PMC10755569; doi:10.1192/j.eurpsy.2023.2457)
Supplement: Rinaldi et al. supplementary material [file S0924933823024574sup001.docx]

# Supplementary Material to:

# Exploring the complex relationships between coping strategies, locus of control and self-esteem with psychopathology: Structural Equation Modeling with a special focus on clinical high risk of psychosis

## Giulia Rinaldi; Naweed Osman; Michael Kaess; Benno G. Schimmelmann; Jochen Kindler; Frauke Schultze-Lutter; Chantal Michel

**Contents: 6 eTexts, 8 eTables, 9 eFigures**

- eText 1: The Bern Epidemiological At-Risk (BEAR) study - details on study design …………3
- eText 2: Details on recruitment of sample …………………………………………………………4
- eFigure 1: Composition of the community sample………………………………………………...6
- eText 3: The Bern Early Recognition and Intervention Centre for mental crisis (FETZ Bern)..7
- eText 4: Details on participants excluded from the clinical sample……………………………...8
- eTable 1: Clinical high-risk symptoms and criteria of first-episode psychosis………………….9
- eText 5: Details regarding assessments used in the present study……………………………11
- eTable 2: Results of the EFA in the community sample………………………………………...13
- eTable 3: Results of the CFA in the community sample…………………………………….....13
- eText 6: Description of the six alternative SEM models………………………………………...14
- eFigure 2: Model 1.1………………………………………………………………………………..15
- eFigure 3: Model 1.2. (chosen as best fitting the community sample data)…………………..15
- eFigure 4: Model 2.1……………………………………………………………………………….16
- eFigure 5: Model 2.2……………………………………………………………………………….16
- eFigure 6: Model 3.1……………………………………………………………………………….17
- eFigure 7: Model 3.2……………………………………………………………………………….17
- eTable 4: Fit indices of the six alternative SEM-models to the community sample………….18
- eTable 5: Standardized regression coefficients (β), covariance coefficients (s) and p values in model 1.2.; community and clinical sample……………………………………………………19
- eFigure 8: Model 1.2., community sample, sensitivity analysis………………………………...20
- eTable 6: Community sample - Standardized regression coefficients (β) and p values for paths in model 1.2., sensitivity analysis…………………………………………………………..21
- eFigure 9: Model 1.2., clinical sample, sensitivity analysis……………………………………..22
- eTable 7: Clinical sample - Standardized regression coefficients (β) and p values for paths in model 1.2., sensitivity analysis…………………………………………………………………….23
- eTable 8: Mediation effect analyses and 95% bias-corrected bootstrap CI, sensitivity analysis in the community and the clinical sample…………………………………………………………24

**eText 1: The Bern Epidemiological At-Risk (BEAR) study - details on study design**

At baseline, a representative sample of the Bernese general population was obtained using a stratified sampling method. Participants were randomly selected from the approximately 310,000 predominantly Caucasian 16 to 40 years old residents of the semi-rural Canton Bern.

The community sample was evaluated during a semi-structured telephone interview. Excellent concordance rates (78-100%) were found for telephone and face-to-face assessment for the used clinical interviews in a feasibility study that was carried out prior to the BEAR-study baseline assessment [1].

Eligibility criteria were inclusion in the selected age range, main residency in Canton Bern (i.e. having a valid address in the Canton and not being abroad during the assessment period), and an available telephone number.

First telephone contact was attempted two weeks after sending eligible participants a one-page information letter, meant to increase response rates, and explaining the study goals and procedure, as well as the incentives for participation.

Participation in the telephone interview after receiving exhaustive information about the study was considered as giving informed consent. Eligible participants that could not be reached after up to 100 calls over several months, at different times and days including Saturdays, were considered as unknown eligible.

Further exclusion criteria were (i) a lifetime diagnosis of psychosis [2] and (ii) insufficient fluency in German, French or English. If respondents met one of these criteria, their interview was interrupted prematurely. On average, the semi-structured interviews lasted 43 minutes (SD: 20 minutes; range: 20–225 minutes).

To ensure an excellent assessment quality, clinical psychologists conducted the telephone interviews after three months of intensive training, and were provided with weekly supervision by F. Schultze-Lutter and C. Michel [2].

**eText 2: BEAR-study - details on recruitment of sample and representativeness**

*Baseline*

Out of 4,471 eligible participants, 2,857 were interviewed. Due to insufficient language skills, 125 (4.4%) interviews were interrupted prematurely; furthermore, 41 (1.4%) interviews were aborted due to a lifetime diagnosis of psychosis (19 of these were not diagnosed/treated) [2] and 8 (0.3%) participants prematurely terminated the interviews themselves. The 1,350 (29.5%) refusers cited lack of time or interest as the main reason for not taking part in the study.

Completed interviews were 2,683, with a contact rate of 94.8% and a response rate of 63.4%. Compared to the 16- to 40-year-old general population of Bern, the eligible sample was negligibly older, but this difference was mainly based on a higher non-significant number of available telephone numbers (landlines) in 36- to 40-year-olds.

For the 2,683 participants who completed the interview, negligible differences were detected in age distribution, but not gender, nationality or marital status, when compared to the 16- to 40-year-old general population of Bern. They were therefore considered to be a representative sample of their age group [3].

*Follow-up*[4]

In their baseline interview, 659 participants (23.1% of the 2,857 interviewed) reported CHR symptoms or criteria; of these, 97.9% (n=645) gave their consent to be re-contacted for a future assessment, thus constituting the main target group (RISK) for the follow-up. Next, a control group (CONTROL) of 645 persons who didn’t report any CHR symptoms or criteria at baseline were selected, after matching them to RISK participants for both (i) gender and (ii) age at baseline. CONTROL subjects that were ineligible or refused to participate were replaced by another match to the respective RISK participant. Including these, 1,263 participants were re-contacted for the follow-up assessment, with a contact rate of 78.8%. In total, 839 interviews were conducted (response rate 66.4%), including 829 non-conversions, 5 conversions to psychosis, and 5 partial interviews.

*The community sample in the present study*

A total of 523 participants in the follow up of the BEAR study were included in the community sample included in the present study. Their main sociodemographic characteristics are described in Table 1. The 109 participants who were not eligible for the add-on study or did not provide their consent to complete the questionnaires (see Figure 1 for further details) were mostly male (63.3%), had a median age of 35 (mean age: 32.9 ± 8) and most (86.2%) had completed at least a short-cycle tertiary education (ISCED level ≥ 5). Those who agreed to complete the questionnaires but did not return them by the end of the study (N = 202) were also mostly male (57.9%), had a median age of 35 (mean age: 33.0 ± 7.6) and most (91.1%) reported a high level of education (ISCED level ≥ 5).

See eFigure 1 for a graphic depiction of the composition of the community sample used in the present study.

**eFigure 1: Composition of the community sample**

**
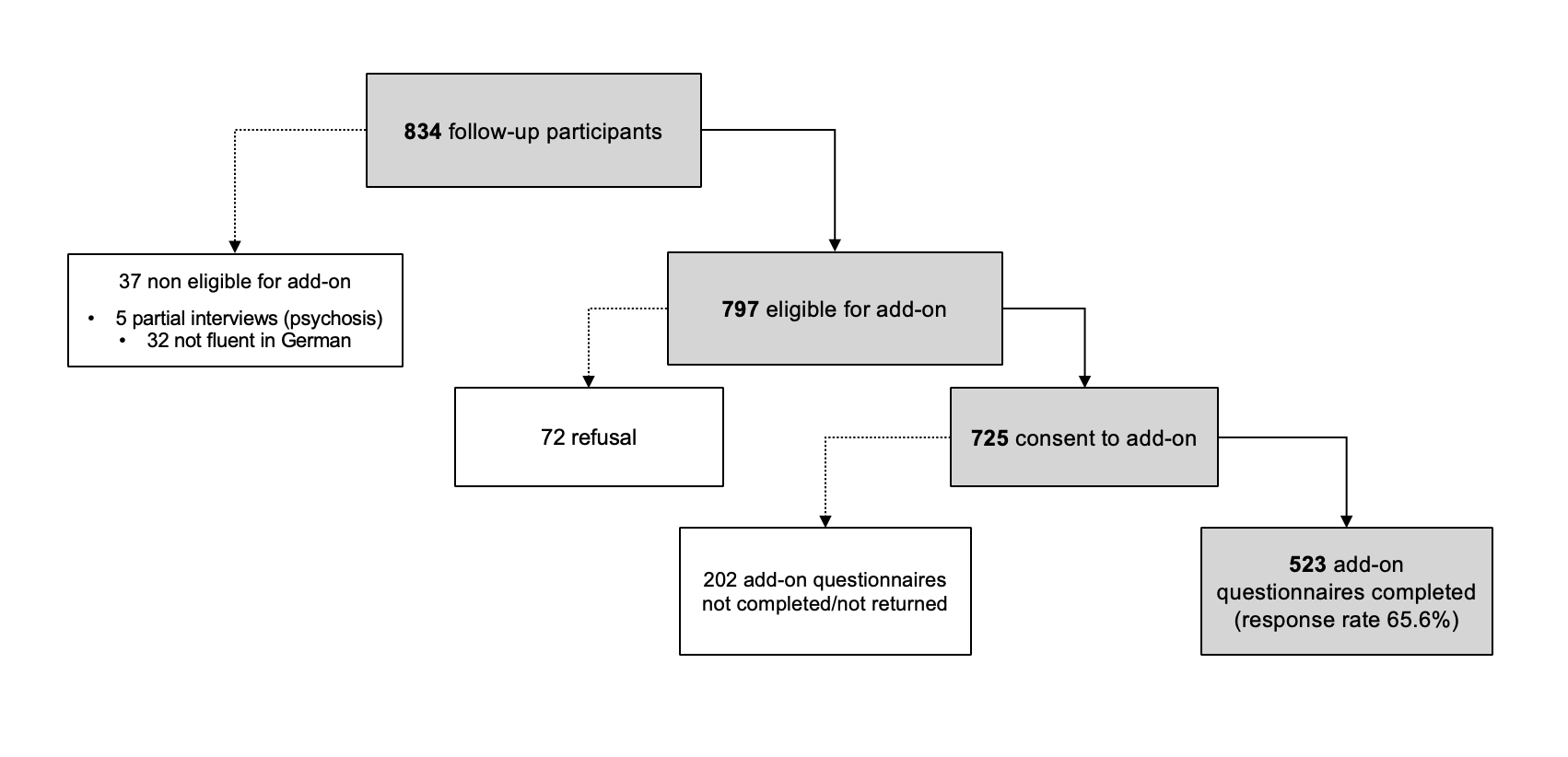
**

Participants in the Bern Epidemiological At Risk (BEAR) study who were included in the community sample used in the present study.

**eText 3: The Bern Early Recognition and Intervention Centre for mental crisis (FETZ Bern)**

The FETZ Bern [5] ([www.upd.ch/fetz](http://www.upd.ch/fetz)) is the only specialized outpatient clinical center for early detection of psychosis in the Canton of Bern, Switzerland, serving an area with 1 million inhabitants. Its target population are help-seeking persons between 8 and 40 years of age with putative psychotic symptoms or CHR symptoms, who are provided with a naturalistic, but close to scientific monitoring of all consecutive referrals. Patients with various psychiatric symptoms are admitted to the FETZ Bern; however, persons with (i) past clinical diagnosis of any psychotic disorder according to DSM and ICD, (ii) diagnosis of delirium, dementia, amnestic or other neurological disorders, and (iii) general medical conditions affecting the central nervous system are excluded from treatment. The diagnostic assessment of CHR symptoms follows the international gold standards for psychosis risk detection [6]. The FETZ Bern works in close cooperation with the outpatient facilities and inpatient units of the University Psychiatry Department Bern (UPD) and the Soteria Bern, allowing for an efficient referral into adequate treatment. The FETZ Bern is also financed by clinical accounting of these institution, along with research funds of the University of Bern.

If there is clinical suspicion of psychotic development, patients can be admitted to the FETZ Bern either of their own initiative or after referral by physicians or psychosocial institutions. Anyone can contact the FETZ Bern via the service phone, email, mail or in person. Before the first appointment, they will then be contacted telephonically by a clinical psychologist for a first assessment of the clinical indication for treatment at the FETZ Bern. This first evaluation covers CHR symptoms, social decline, genetic risk and drug abuse, and, when this indication is not met, it leads to referral into appropriate diagnostics or treatment. Three clinical psychologists, supervised by the clinical head psychologist and board-certified psychiatrists, conduct all assessments. Administrative processes are carried out with the aid of an assistant clinical psychologist and an intern. Patients and, for minors, their legal guardians provide their informed consent for use of their anonymized clinical data in scientific analyses and publications, as per requirement of the local ethics committee (ID PB_2016-01991).

**eText 4: Details on participants excluded from the clinical sample**

The clinical sample included in the present study consisted of 378 participants.

Fifty-one participants were excluded from the analyses due to having more than 50% missing data. They were 50.1% male (N = 26) and had a median age of 17.21 years (mean age: 19.07 ±4.52). Further, 35.3% of them (N = 18) reported a high level of education (ISCED level ≥ 5), while 31.4% (N = 16) were missing this information, 11.8% (N = 6) had completed high school or high school-level professional education (ISCED level: 3.4/3.5), 19.6% (N = 10) secondary school (ISCED level: 2), and one person (1.96%) had finished primary school only (ISCED level: 1).

**eTable 1: Clinical high-risk symptoms and criteria of first-episode psychosis**

| **Ultra-high risk (UHR) criteria** according to the SIPS |
| --- |
| A. ‘Brief Intermittent Psychotic Symptoms’ (BIPS)  ⮊ At least any 1 of the following SIPS P-items scored 6 ‘severe and psychotic’   - P1 Unusual Thought Content / Delusional Ideas - P2 Suspiciousness / Persecutory Ideas - P3 Grandiose Ideas - P4 Perceptual Abnormalities / Hallucinations - P5 Disorganized Communication   ⮊ First appearance in the past three months  ⮊ Present for at least several minutes per day at a frequency of at least once per month but less than 7 days |
| B. ‘Attenuated Positive Symptoms’ (APS)  ⮊ At least any 1 of the following SIPS P-items scored 3 ‘moderate’ to 5 ‘severe but not psychotic’   - P1 Unusual Thought Content / Delusional Ideas - P2 Suspiciousness / Persecutory Ideas - P3 Grandiose Ideas - P4 Perceptual Abnormalities / Hallucinations - P5 Disorganized Communication   ⮊ First appearance within the past year or current rating one or more scale points higher compared to 12 months ago  ⮊ Symptoms have occurred at an average frequency of at least once per week in the past month |
| C. ‘Genetic Risk and Deterioration’ Syndrome  (1) Patient meets criteria for Schizotypal Personality Disorder according to SIPS  (2) Patient has 1^st^ degree relative with a psychotic disorder  (3) Patient has experienced >30% drop in global assessment of functioning (GAF) score over the last month compared to 12 months ago  ⮊ [1 and 3] or [2 and 3] or all are met. |
| **Basic symptom criteria** |
| Risk criterion ‘Cognitive-Perceptive Basic Symptoms’ (COPER)  ⮊ At least any 1 of the following basic symptoms with a SPI-A score of ≥3 within the last 3 months:   - Thought interference - Thought perseveration - Thought pressure - Thought blockages - Disturbance of receptive speech - Decreased ability to discriminate between ideas and perception, fantasy and true memories - Unstable ideas of reference - Derealisation - Visual perception disturbances (excluding hypersensitivity to light or blurred vision) - Acoustic perception disturbances (excluding hypersensitivity to sounds)   ⮊ First occurrence ≥12 months ago |
| High-risk criterion ‘Cognitive Disturbances’ (COGDIS)  ⮊ At least any 2 of the following basic symptoms with a SPI-A score of ≥3 within the last 3 months:   - Inability to divide attention - Thought interference - Thought pressure - Thought blockages - Disturbance of receptive speech - Disturbance of expressive speech - Unstable ideas of reference - Disturbances of abstract thinking - Captivation of attention by details of the visual field |

**eText 5: Details regarding assessments used in the present study**

*Mini-International Neuropsychiatric Interview* (MINI)

The Mini-International Neuropsychiatric Interview [7] was used to assess current present of following mental disorders according to the Diagnostic and Statistical Manual of Mental Disorders, Fourth Edition (DSM-IV) [8] criteria: anxiety and mood disorders, depressive and (hypo-)manic episodes, obsessive-compulsive disorders, post-traumatic stress disorders, substance dependence/abuse, eating disorders, somatization disorders, hypochondriasis, body dysmorphic and pain disorders.

*EuroQoL-5D, 3 level version* (EQ-5D-3L)

The EQ-5D-3 L [9] sum score we used in our analyses was obtained via the following formula by Hinz and colleagues [9] (see also [10]):

(100-(10x [value1+value2+value3+value4+value5-5]))

The 5 values refer to the 5 dimensions of mobility, self-care, usual activities, pain/discomfort, anxiety/depression, self-rated on 3 degrees of severity (from absence of problems to extreme difficulties).

*German Competence and Control Beliefs Questionnaire* (FKK)

In our analyses, we used three scales from the German Competence and Control Beliefs Questionnaire (FKK) [11].

Two were primary scales, each with a sum score obtained from 8 corresponding items.

We used the Self-Efficacy primary scale (FKK-SK), referring to the positive self-concept of one’s own competencies, to represent competence beliefs, as indicated in the instrument’s manual [11].

To indicate adaptive LOC, we used the Internality primary scale (FKK-I), assessing the tendency to a general attribution of control/causality to the self in relation to life events. This is coherent with the conceptualization of adaptive LOC as ‘internal LOC’, or, alternatively, as ‘internality’, as originally defined in Rotter’s social learning theory [12], on which the FKK is based.

The third was a secondary scale, the Externality scale (FKK-PC), obtained from the aggregation of two primary scales, and evaluating the tendency to a general attribution of control on life events to fatalistic (Fatalistic Externality primary scale, FKK-C) and/or social causality (Social Externality primary scale, FKK-P). This scale was used to conceptualize maladaptive LOC, defined as ‘external LOC’, or, alternatively, as ‘externality’, by Rotter [12]. The FKK-PC score is calculated by summing the 16 items that form the FKK-P and FKK-C primary scales. The choice of employing two primary (FKK-I, FKK-SK) and one secondary (FKK-PC) scale to conceptualize core beliefs in our SEM models, instead of scales on the same level of complexity, was meant to reflect the concepts and model structure resulting from the meta-analysis by Groth et al. [13], separating adaptive and maladaptive LOC from competence beliefs.

**eTable 2: Results of the EFA in the community sample**

| EFA results, community sample (N=522) | | | | | | |  |
| --- | --- | --- | --- | --- | --- | --- | --- |
|  | | **Factor loadings** | **Variance explained** | | | |  |
| Psychopathology 68 % | | | | | | |  |
|  | Current axis-I disorders | 0.51 | |  |  |  | |
|  | GAF score | 1.01 | |  |  |  | |
|  | SOFAS score | 0.82 | |  |  |  | |
|  | **Self-rated health** |  | |  | 32% |  | |
|  | EQ-5D summary score | 0.83 | |  |  |  | |
|  | EQ-5D analogue score | 0.44 | |  |  |  | |

**eTable 3: Results of the CFA in the community sample**

| CFA results, community sample (N=522) | | | | | | | | |  |
| --- | --- | --- | --- | --- | --- | --- | --- | --- | --- |
|  | | **Factor loadings** | |  | | |  |  |  |
|  | | **Unstandardized (SD)** | **Standardized** | | |  | |  | |
| Psychopathology | | | | | | |  |  |  |
|  | Current axis-I disorders | 1.00^+^ | 0.736 |  |  | | | | |
|  | GAF score | -20.66 (0.99) | -0.943 |  |  | | | | |
|  | SOFAS score | -13.01 (0.65) | -0.877 |  |  | | | | |
|  | **Self-rated health** |  |  |  |  | | | | |
|  | EQ-5D analogue score | 1.00^+^ | 0.704 |  |  | | | | |
|  | EQ-5D summary score | 0.71 (0.07) | 0.814 |  |  | | | | |

*Note:* +: fixed parameter

**eText 6: Description of the six alternative SEM models**

In all 6 alternative SEM models we tested in the present study:

- age and education (ISCED level) are exogenous variables, while all others are endogenous;
- following Groth and colleagues [13], Positive and Negative Coping Strategies (SVF) play a mediating role in the relationship between competence beliefs (FKK-SK), adaptive (FKK-I) and maladaptive LOC (FKK-PC), while mental health outcomes are represented by the latent factors PP and SRH obtained through the preliminary EFA and CFA.

The models can be further described as follows:

- Models 1.1. and 1.2.: presence of any BS symptom and presence of any UHR symptom (1.1.), or presence of any CHR symptom (1.2.) are associated with higher Psychopathology and lower Self-Rated Health (outcome variables), respectively; they are in turn predicted by competence beliefs and locus of control, and this association is mediated by coping.
- Models 2.1. and 2.2.: presence of any BS symptom and presence of any UHR symptom (2.1.), or presence of any CHR symptom (2.2.), respectively, are outcome variables, parallel to Psychopathology and Self-Rated Health.
- Models 3.1. and 3.2.: presence of any BS symptom and presence of any UHR symptom (3.1.), or presence of any CHR symptom (3.2.), respectively, are outcome variables, influenced by Psychopathology and Self-Rated Health.

For graphic representations of the models, see eFigures 1-6.

**eFigure 2 – Model 1.1.
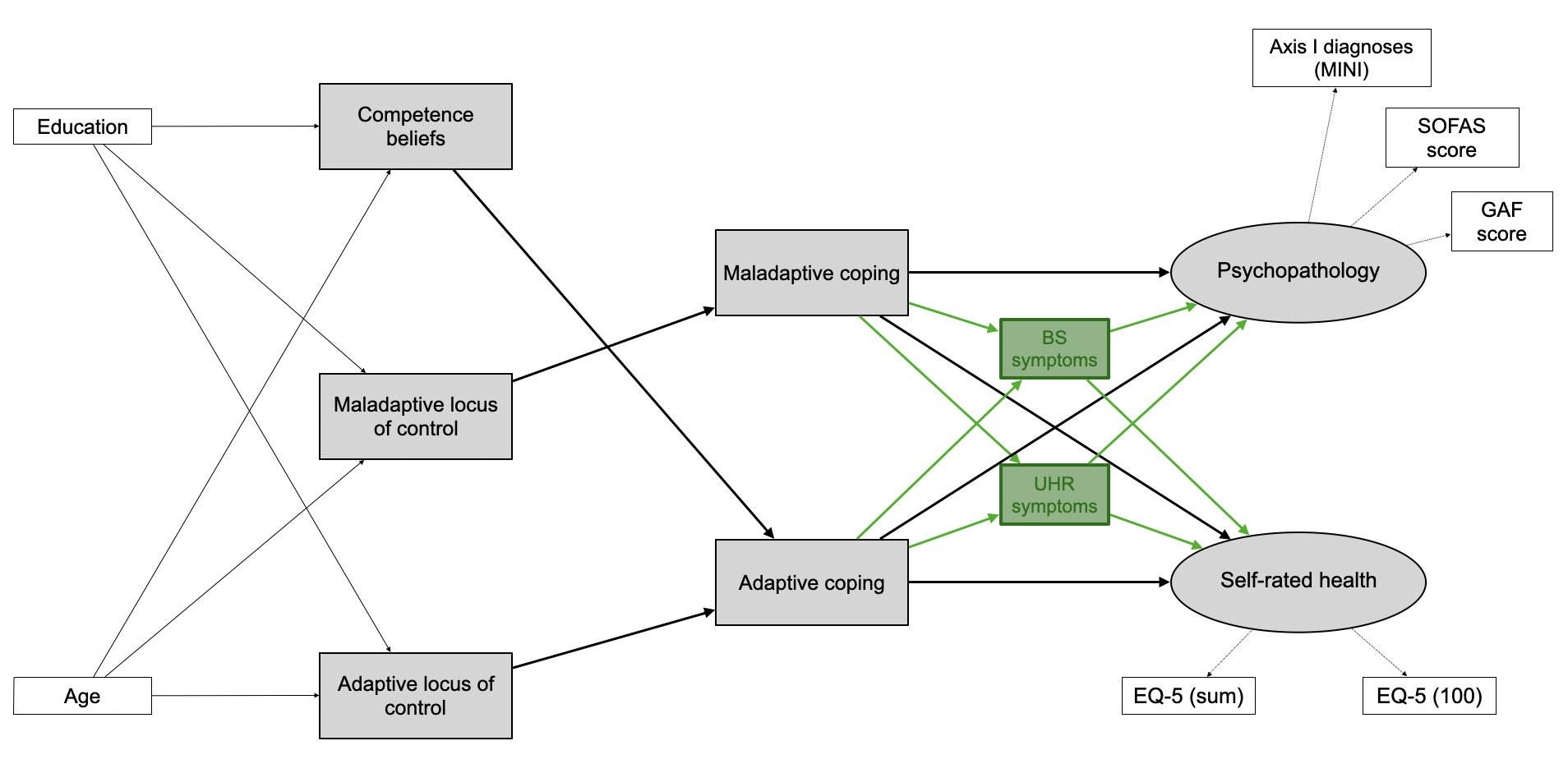
**

*Note:* CHR symptoms: Clinical High-Risk symptoms; EQ-5 (100): score on the 0-100 analogue scale of the EuroQoL-5D, 3 level version (EQ-5D-3L) analogue scale; EQ-5 (sum): sum score on the EQ-5D-3L – see eText 5 for details; SOFAS score: Social and Occupational Functioning Scale score; GAF score: Global Assessment of Functioning score; MINI: Mini-International Neuropsychiatric Interview.

**eFigure 3 – Model 1.2. (chosen as best fitting the community sample data)**

**
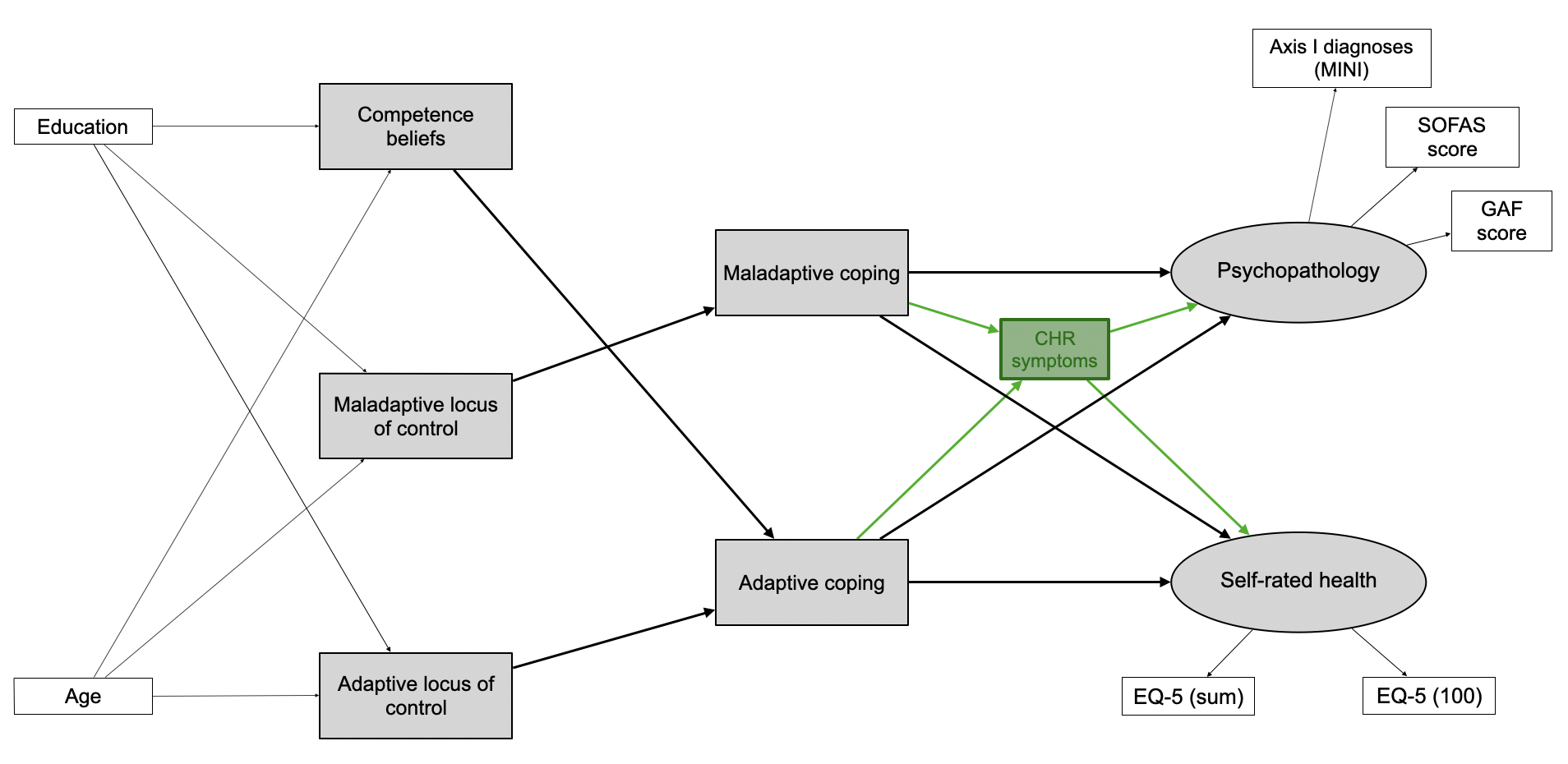
**

*Note:* CHR symptoms: Clinical High-Risk symptoms; EQ-5 (100): score on the 0-100 analogue scale of the EuroQoL-5D, 3 level version (EQ-5D-3L) analogue scale; EQ-5 (sum): sum score on the EQ-5D-3L – see eText 5 for details; SOFAS score: Social and Occupational Functioning Scale score; GAF score: Global Assessment of Functioning score; MINI: Mini-International Neuropsychiatric Interview.

**eFigure 4 – Model 2.1.**

**
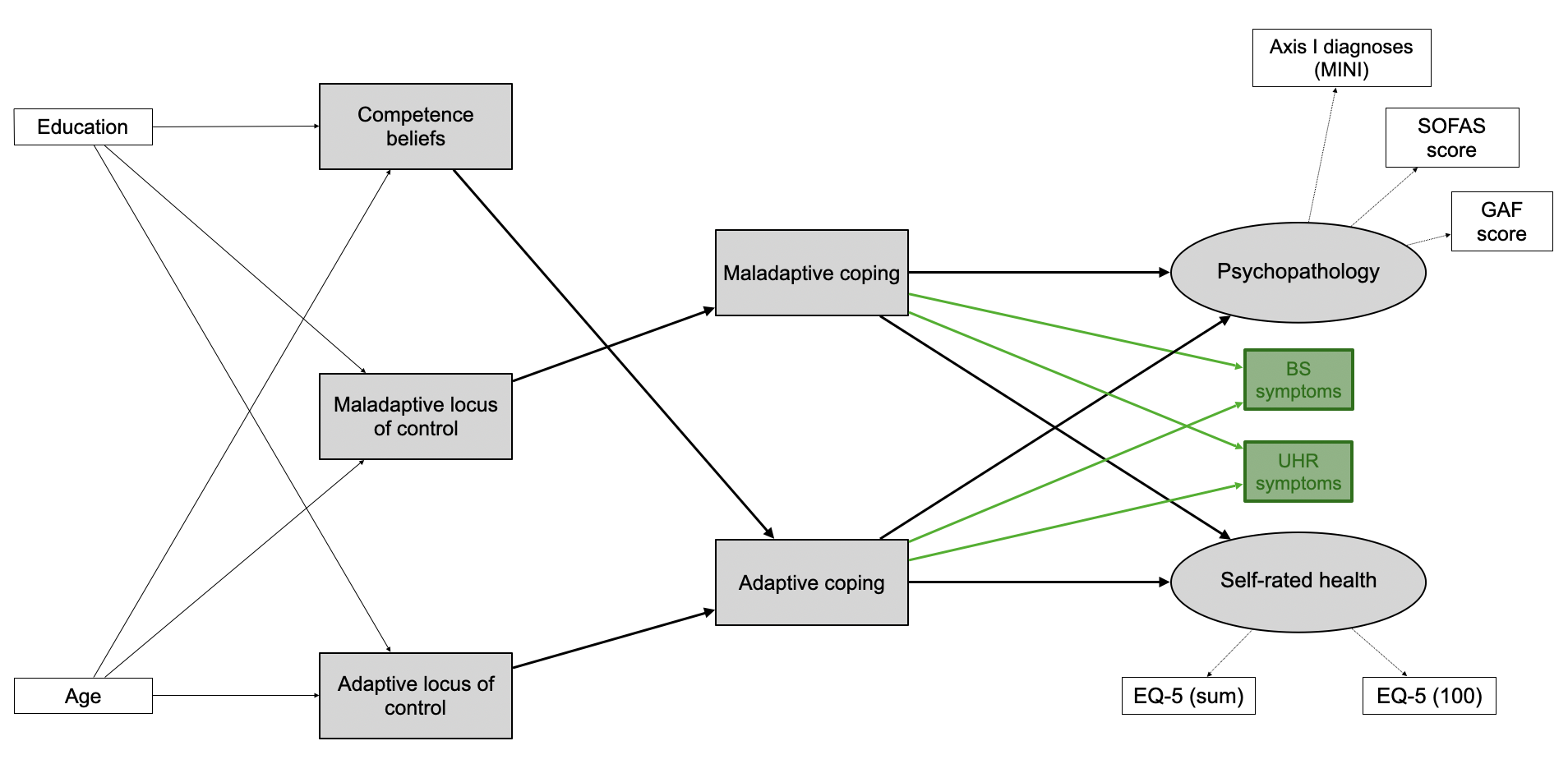
**

*Note:* CHR symptoms: Clinical High-Risk symptoms; EQ-5 (100): score on the 0-100 analogue scale of the EuroQoL-5D, 3 level version (EQ-5D-3L) analogue scale; EQ-5 (sum): sum score on the EQ-5D-3L – see eText 5 for details; SOFAS score: Social and Occupational Functioning Scale score; GAF score: Global Assessment of Functioning score; MINI: Mini-International Neuropsychiatric Interview.

**eFigure 5 – Model 2.2.**

**
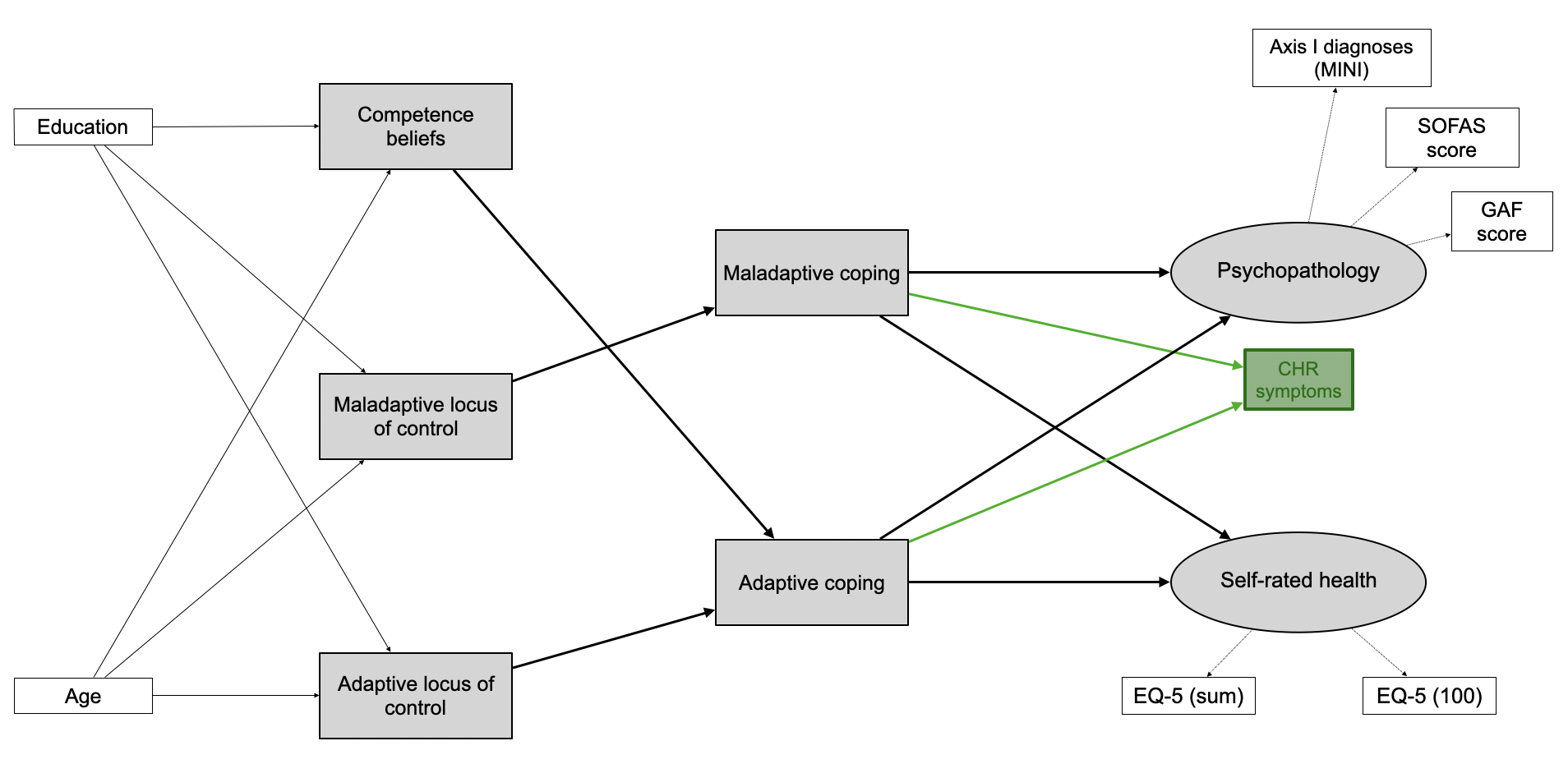
**

*Note:* CHR symptoms: Clinical High-Risk symptoms; EQ-5 (100): score on the 0-100 analogue scale of the EuroQoL-5D, 3 level version (EQ-5D-3L) analogue scale; EQ-5 (sum): sum score on the EQ-5D-3L – see eText 5 for details; SOFAS score: Social and Occupational Functioning Scale score; GAF score: Global Assessment of Functioning score; MINI: Mini-International Neuropsychiatric Interview.

**eFigure 6 – Model 3.1.**

**
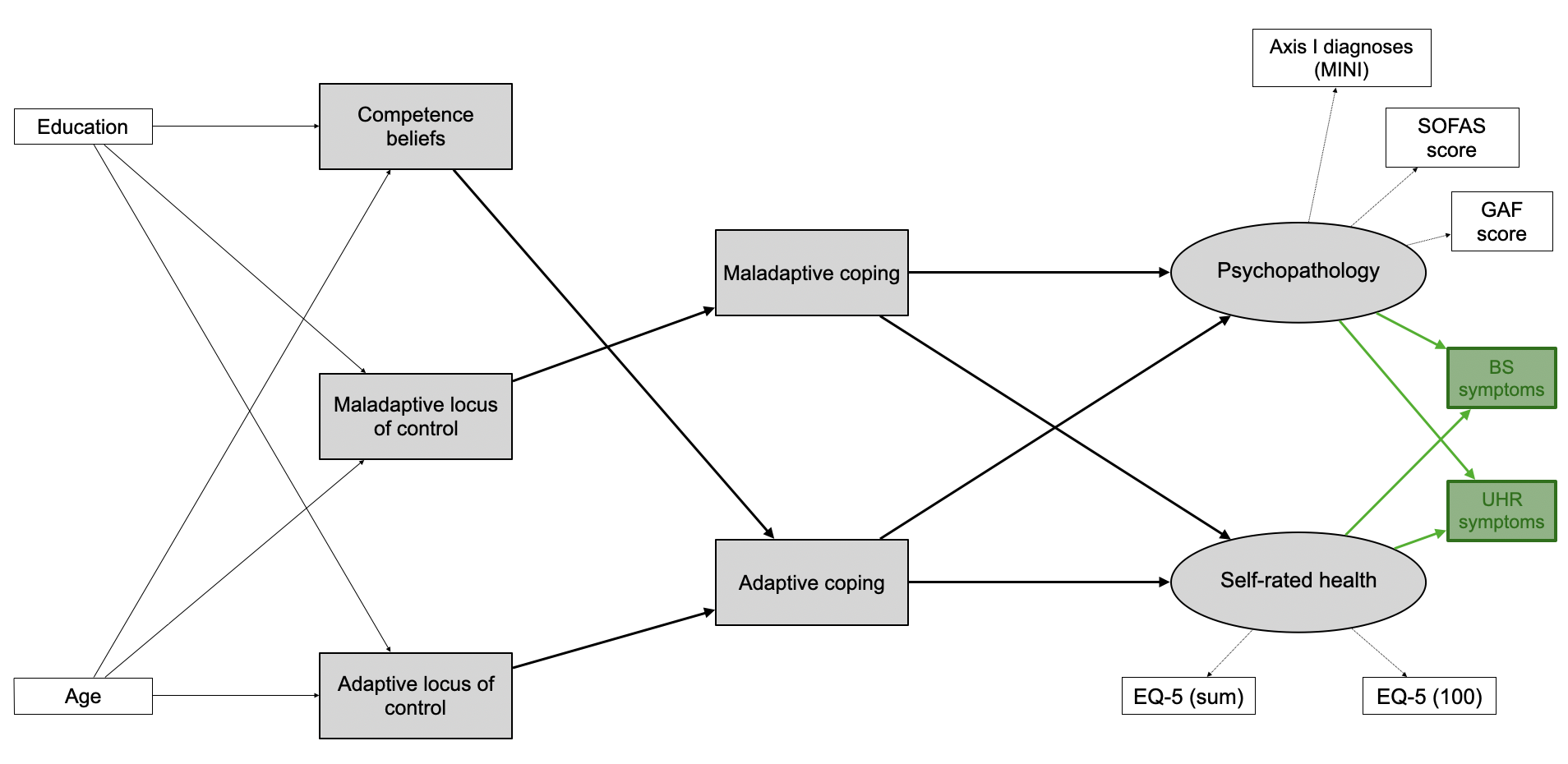
**

*Note:* CHR symptoms: Clinical High-Risk symptoms; EQ-5 (100): score on the 0-100 analogue scale of the EuroQoL-5D, 3 level version (EQ-5D-3L) analogue scale; EQ-5 (sum): sum score on the EQ-5D-3L – see eText 5 for details; SOFAS score: Social and Occupational Functioning Scale score; GAF score: Global Assessment of Functioning score; MINI: Mini-International Neuropsychiatric Interview.

**eFigure 7 – Model 3.2.**

**
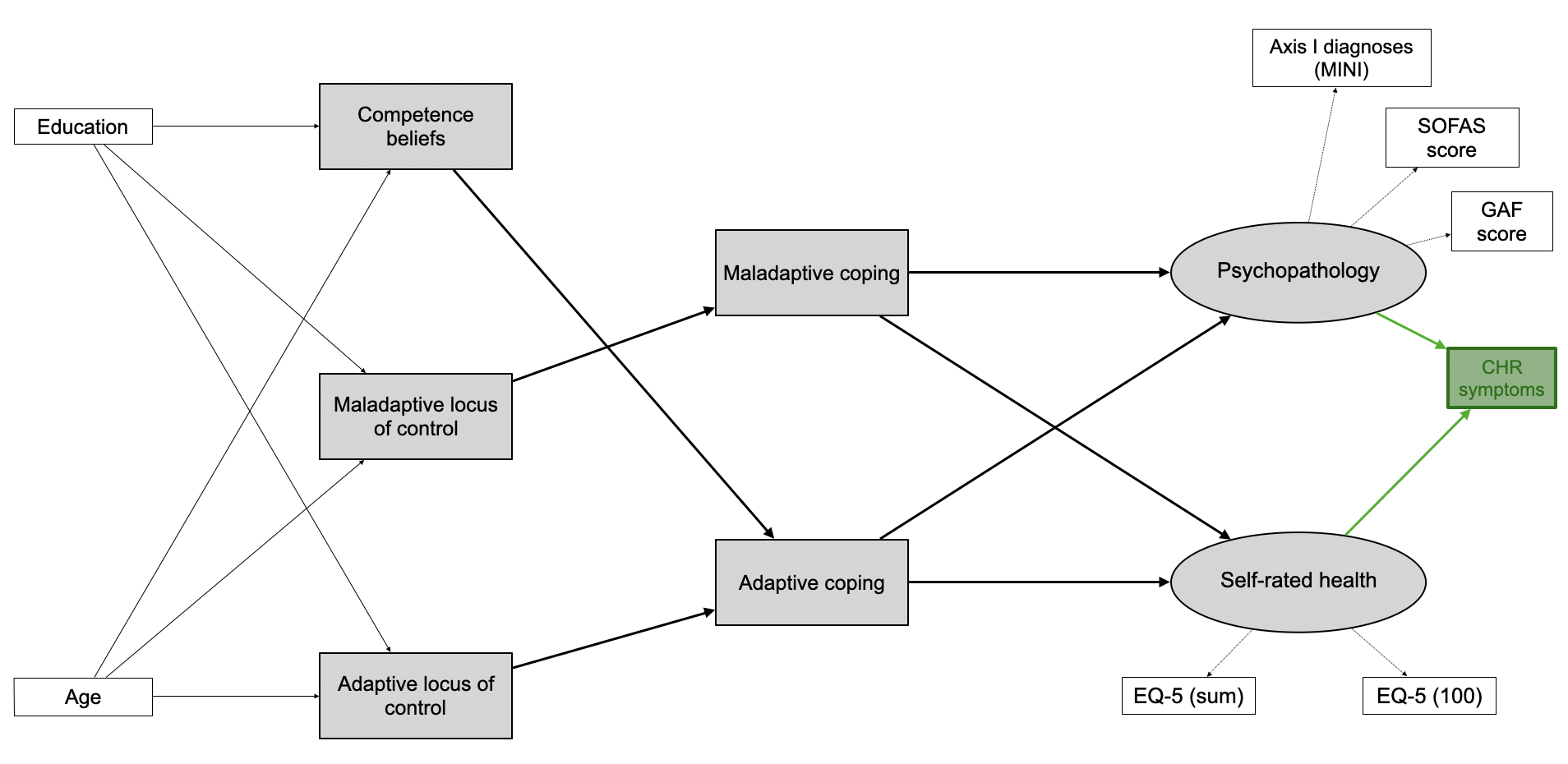
**

*Note:* CHR symptoms: Clinical High-Risk symptoms; EQ-5 (100): score on the 0-100 analogue scale of the EuroQoL-5D, 3 level version (EQ-5D-3L) analogue scale; EQ-5 (sum): sum score on the EQ-5D-3L – see eText 5 for details; SOFAS score: Social and Occupational Functioning Scale score; GAF score: Global Assessment of Functioning score; MINI: Mini-International Neuropsychiatric Interview.

**eTable 4: Fit indices of the six alternative SEM-models to the community sample**

| ***Model*** | **CFI** | **TLI** | **RMSEA** | **90%CIs** | **SRMR** | **AIC** | **BIC** |
| --- | --- | --- | --- | --- | --- | --- | --- |
| ***1.1.*** | 0,926** | *0,871* | 0,078** | 0.067-  0.089* | 0,052*** | 39735,789 | 39961,037 |
| ***1.2.*** | 0,923** | *0,863* | 0,086* | 0.075-  0.098* | 0,055*** | **39484,669** | 39684,418 |
| ***2.1.*** | 0.926** | *0,865* | 0,080** | 0.069-  0.091* | 0,051*** | 39739,031 | 39972,780 |
| ***2.2.*** | 0,922** | *0,859* | 0,088* | 0.076-0.100* | 0,055*** | 39486,477 | 39690,476 |
| ***3.1.*** | 0,923** | *0,875* | 0,077** | 0.067-0.088* | 0,055*** | 39739,283 | 39947,532 |
| ***3.2.*** | 0,921** | *0,867* | 0.085* | 0.074-0.097* | 0.057*** | 39485,825 | **39677,074** |

*Note:* * = acceptable fit; ** = good fit; *** = excellent fit. Values in cursive represent poor fit to the data. The best, i.e., lowest AIC and BIC values are in bold.

CFI (comparative fit index) is considered excellent if >0.95, good if >0.90, poor if <0.90;

TLI (Tucker-Lewis index) is considered excellent if >0.95, good if >0.90, poor if <0.90;

RMSEA (root-mean-square error of approximation) is considered excellent if <0.60, good if 0.06-0.08, acceptable if 0.08-0.10. poor if >0.10;

90%CI (confidence interval) is considered excellent if it does not include 0.08, good if relatively narrow (e.g. 0.70 to 0.80), acceptable if relatively wide (e.g. 0.60 to 0.93), poor if very wide (e.g.0.50 to 0.10);

SRMR (standardized root mean square residual) is considered excellent if <0.08, good if 0.08-0.10, poor if >0.10.

**eTable 5: Standardized regression coefficients (β), covariance coefficients (s) and p values in model 1.2.; community and clinical sample**

| Model 1.2., community sample (N=518) | | | | | | Model 1.2., clinical sample (N=327) | | |
| --- | --- | --- | --- | --- | --- | --- | --- | --- |
|  | | **β** | **p** | | | | **β p** | |
| Psychopathology (PP) | | | | | | |  | |
|  | Maladaptive coping | 0.236 | |  | <0.001** | | *-0.053* | *0.401* |
|  | Adaptive coping | -0.108 | |  | 0.009* | | *-0.080* | *0.212* |
|  | CHR symptoms | 0.358 | |  | <0.001** | | 0.313 | <0.001** |
|  | Competence beliefs | *-0.033* | |  | *0.518* | | *-0.122* | *0.083* |
|  | Maladaptive LOC | *0.081* | |  | *0.111* | | *0.041* | *0.548* |
|  | Adaptive LOC | *-0.042* | |  | *0.361* | | *0.039* | *0.538* |
|  | **Maladaptive coping** |  | |  |  | |  |  |
|  | Maladaptive LOC | 0.525 | |  | <0.001** | | 0.433 | <0.001** |
|  | **Adaptive coping** |  | |  |  | |  |  |
|  | Competence beliefs | 0.188 | |  | <0.001** | | 0.275 | <0.001** |
|  | Adaptive LOC | 0.171 | |  | <0.001** | | 0.266 | <0.001** |
|  | **Self-rated health (SRH)** |  | |  |  | |  |  |
|  | Maladaptive coping | -0.201 | |  | 0.001** | | *-0.007* | *0.927* |
|  | CHR symptoms | -0.185 | |  | <0.001** | | -0.434 | <0.001** |
|  | Competence beliefs | *-0.030* | |  | *0.636* | | 0.230 | 0.004* |
|  | Adaptive coping | *0.060* | |  | *0.239* | | *0.003* | *0.971* |
|  | Maladaptive LOC | *-0.076* | |  | *0.235* | | *0.066* | *0.395* |
|  | Adaptive LOC | *0.088* | |  | *0.126* | | *-0.030* | *0.673* |
|  | **CHR symptoms** |  | |  |  | |  |  |
|  | Adaptive coping | *-0.003* | |  | *0.947* | | -0.153 | 0.005* |
|  | Maladaptive coping | 0.223 | |  | <0.001** | | 0.204 | <0.001** |
|  | **Competence beliefs** |  | |  |  | |  |  |
|  | ISCED level | 0.188 | |  | <0.001** | | *0.101* | *0.113* |
|  | age | *0.082* | |  | *0.060* | | *-0.123* | *0.054* |
|  | **Adaptive LOC** |  | |  |  | |  |  |
|  | ISCED level | 0.135 | |  | 0.002* | | *-0.020* | *0.756* |
|  | age | *-0.043* | |  | *0.333* | | *-0.004* | *0.948* |
|  | **Maladaptive LOC** |  | |  |  | |  |  |
|  | ISCED level | -0.128 | |  | 0.004* | | *-0.092* | *0.150* |
|  | age | -0.133 | |  | 0.010* | | *0.063* | *0.323* |
|  |  | **s** | |  | **P** | | **s** | **P** |
|  | PP - SRH | -0.474 | |  | <0.001** | | -0.378 | <0.001** |
|  | Adaptive coping – maladaptive coping | *0.011* | |  | *0.811* | | *-0.066* | *0.237* |
|  | Competence beliefs – adaptive LOC | 0.502 | |  | <0.001** | | 0.364 | <0.001** |
|  | Competence beliefs – maladaptive LOC | -0.483 | |  | <0.001** | | -0.446 | <0.001** |
|  | Adaptive LOC – maladaptive LOC | -0.233 | |  | <0.001** | | -0.130 | 0.021* |
|  | Age – ISCED level | 0.166 | |  | <0.001** | | 0.495 | <0.001** |

*Note:* ** = p < .001; * = p < .05; *italics*: not significant

**eFigure 8: Community sample, sensitivity analysis**

**
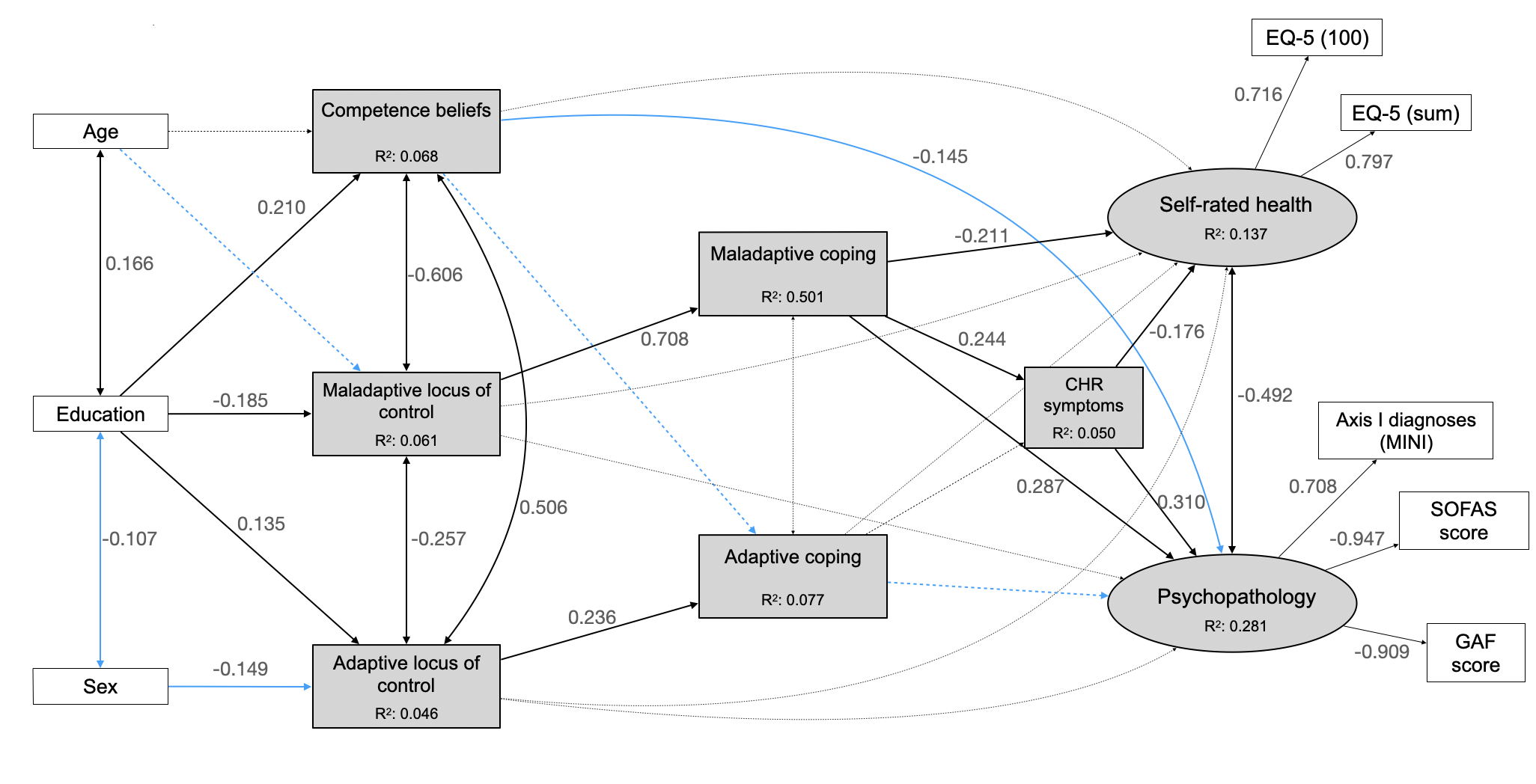
**

*Note:* rectangles represent observed variables, ovals represent unobserved latent variables; black lines with double-ended arrows represent covariances; black lines with single-ended arrows represent significant paths; dashed grey lines with double- or single-ended arrows represent non-significant covariances or regression paths, respectively; numbers next to the lines indicate coefficients of significant standardized regressions and covariances, or factor loadings; the coefficients of non-significant covariances and regressions are not reported here to facilitate the figure’s interpretation; blue arrows represent differences from the original analyses in the community sample. CHR symptoms: Clinical High-Risk symptoms; EQ-5 (100): score on the 0-100 analogue scale of the EuroQoL-5D, 3 level version (EQ-5D-3L) analogue scale; EQ-5 (sum): sum score on the EQ-5D-3L – see eText 5 for details; SOFAS score: Social and Occupational Functioning Scale score; GAF score: Global Assessment of Functioning score; MINI: Mini-International Neuropsychiatric Interview.

Model fit indices: CFI = 0.989, TLI = 0.982, RMSEA = 0.04, SRMR = 0.045

**eTable 6: Community sample** **- Standardized regression coefficients (β) and p values for paths in model 1.2., sensitivity analysis**

| Model 1.2., community sample, sensitivity analysis (N=518) | | | | | | |  |
| --- | --- | --- | --- | --- | --- | --- | --- |
|  | | **β** | | **p** | | |  |
| Psychopathology (PP) | | | | | | |  |
|  | Maladaptive coping | 0.287 | <0.001** | |  |  | |
|  | *Adaptive coping* | *-0.085* | *0.053* | |  |  | |
|  | CHR symptoms | 0.310 | <0.001** | |  |  | |
|  | Competence beliefs | - 0.145 | 0.033* | |  |  | |
|  | **Maladaptive coping** |  |  | |  |  | |
|  | Maladaptive LOC | 0.708 | <0.001** | |  |  | |
|  | **Adaptive coping** |  |  | |  |  | |
|  | *Competence beliefs* | *0.069* | *0.169* | |  |  | |
|  | Adaptive LOC | 0.236 | <0.001** | |  |  | |
|  | **Self-rated health (SRH)** |  |  | |  |  | |
|  | Maladaptive coping | -0.211 | 0.012* | |  |  | |
|  | CHR symptoms | -0.176 | <0.001** | |  |  | |
|  | **Competence beliefs** |  |  | |  |  | |
|  | ISCED level | 0.210 | <0.001** | |  |  | |
|  | **Adaptive LOC** |  |  | |  |  | |
|  | ISCED level | 0.135 | 0.003* | |  |  | |
|  | Sex | -0.149 | 0.005* | |  |  | |
|  | **Maladaptive LOC** |  |  | |  |  | |
|  | ISCED level | -0.185 | <0.001** | |  |  | |
|  | *age* | *-0.085* | *0.090* | |  |  | |
|  |  | **s** | **P** | |  |  | |
|  | PP - SRH | -0.492 | <0.001** | |  |  | |
|  | Competence beliefs – adaptive LOC | 0.506 | <0.001** | |  |  | |
|  | Competence beliefs – maladaptive LOC | -0.606 | <0.001** | |  |  | |
|  | Adaptive LOC – maladaptive LOC | -0.257 | <0.001** | |  |  | |
|  | Age – ISCED level | 0.166 | <0.001** | |  |  | |
|  | ISCED level - sex | -0.107 | 0.049* | |  |  | |

*Note:* ** = p < .001; * = p < .05; *italics*: not significant in the sensitivity analysis

**eFigure 9: Model 1.2., clinical sample, sensitivity analysis**


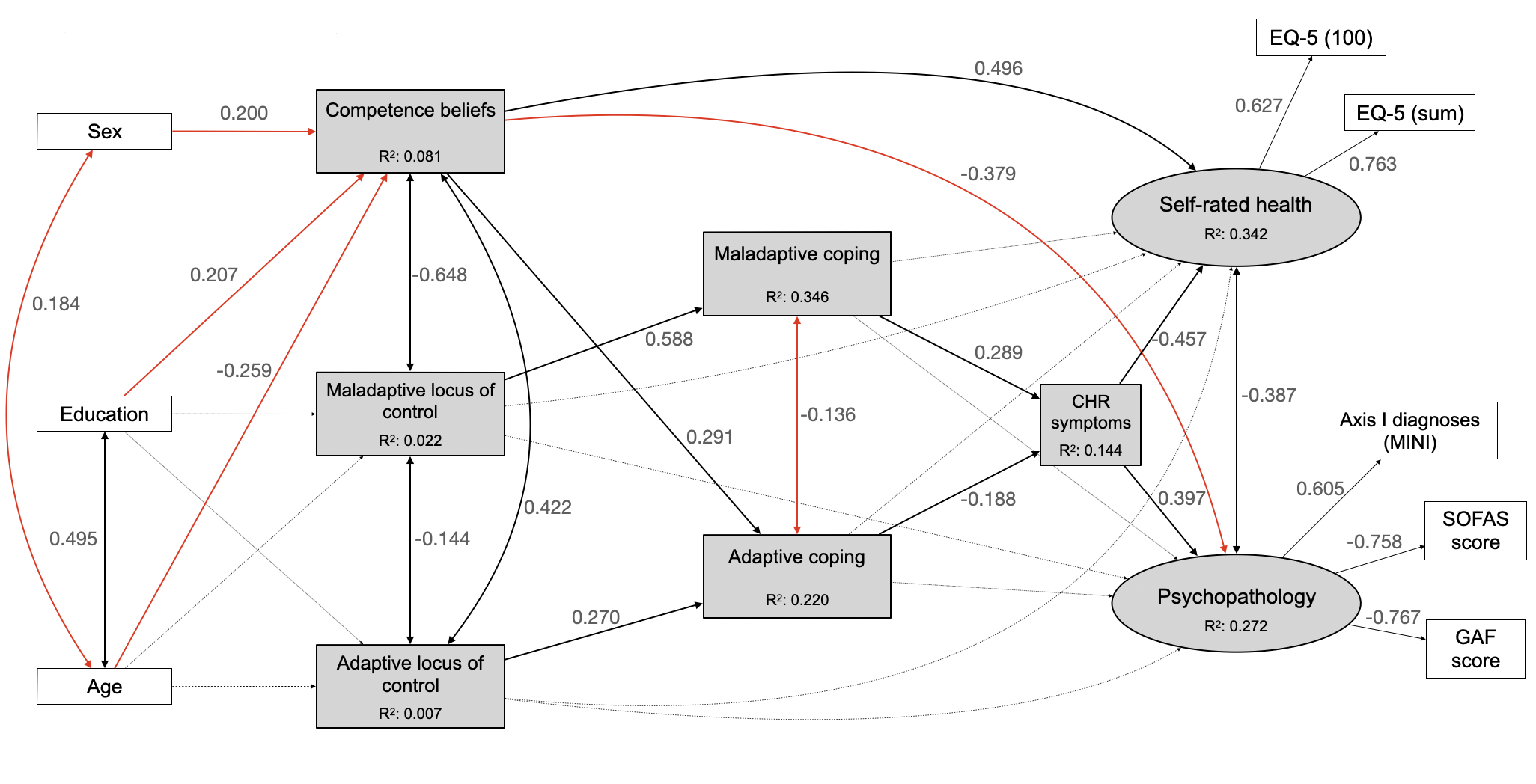


*Note*: rectangles represent observed variables, ovals represent unobserved latent variables; black lines with double-ended arrows represent covariances; black lines with single-ended arrows represent significant paths; dashed grey lines with double- or single-ended arrows represent non-significant covariances or regression paths, respectively; numbers next to the lines indicate coefficients of significant standardized regressions and covariances, or factor loadings; the coefficients of non-significant covariances and regressions are not reported here to facilitate the figure’s interpretation; red arrows represent differences from the original analyses in the clinical sample. CHR symptoms: Clinical High-Risk symptoms; EQ-5 (100): score on the 0-100 analogue scale of the EuroQoL-5D, 3 level version (EQ-5D-3L) analogue scale; EQ-5 (sum): sum score on the EQ-5D-3L – see eText 5 for details; SOFAS score: Social and Occupational Functioning Scale score; GAF score: Global Assessment of Functioning score; MINI: Mini-International Neuropsychiatric Interview.

Model fit indices: CFI = 0.942, TLI = 0.898, RMSEA= 0.068, SRMR = 0.068

**eTable 7: Clinical sample - Standardized regression coefficients (β) and p values for paths in model 1.2., sensitivity analysis**

| Model 1.2., clinical sample, sensitivity analysis (N=327) | | | | | | |  |
| --- | --- | --- | --- | --- | --- | --- | --- |
|  | | **β** | | **p** | | |  |
| Psychopathology (PP) | | | | | | |  |
|  | *Maladaptive coping* | *-0.069* | *0.416* | |  |  | |
|  | *Adaptive coping* | *-0.037* | *0.601* | |  |  | |
|  | CHR symptoms | 0.397 | <0.001** | |  |  | |
|  | Competence beliefs | -0.379 | 0.002* | |  |  | |
|  | **Self-rated health (SRH)** |  |  | |  |  | |
|  | *Maladaptive coping* | *-0.042* | *0.698* | |  |  | |
|  | CHR symptoms | -0.457 | <0.001** | |  |  | |
|  | Competence beliefs | 0.496 | <0.001** | |  |  | |
|  | **CHR symptoms** |  |  | |  |  | |
|  | Adaptive coping | -0.188 | 0.003* | |  |  | |
|  | Maladaptive coping | 0.289 | <0.001** | |  |  | |
|  | **Maladaptive coping** |  |  | |  |  | |
|  | Maladaptive LOC | 0.588 | <0.001** | |  |  | |
|  | **Adaptive coping** |  |  | |  |  | |
|  | Competence beliefs | 0.291 | <0.001** | |  |  | |
|  | Adaptive LOC | 0.270 | <0.001** | |  |  | |
|  | **Competence beliefs** |  |  | |  |  | |
|  | ISCED level | 0.207 | 0.012* | |  |  | |
|  | Age | -0.259 | 0.001* | |  |  | |
|  | Sex | -0.200 | 0.019* | |  |  | |
|  | **Adaptive LOC** |  |  | |  |  | |
|  | *ISCED level* | *-0.066* | *0.315* | |  |  | |
|  | **Maladaptive LOC** |  |  | |  |  | |
|  | *ISCED level* | *-0.118* | *0.098* | |  |  | |
|  | *age* | *0.067* | *0.280* | |  |  | |
|  |  | **s** | **P** | |  |  | |
|  | PP - SRH | -0.387 | 0.001** | |  |  | |
|  | Adaptive coping – maladaptive coping | -0.136 | <0.001** | |  |  | |
|  | Competence beliefs – adaptive LOC | 0.422 | <0.001** | |  |  | |
|  | Competence beliefs – maladaptive LOC | -0.648 | <0.001** | |  |  | |
|  | Adaptive LOC – maladaptive LOC | -0.144 | 0.002* | |  |  | |
|  | Age – ISCED level | 0.495 | <0.001** | |  |  | |
|  | Age - sex | -0.184 | 0.009* | |  |  | |
|  | ISCED level - sex | *-0.037* | *0.602* | |  |  | |

*Note:* ** = p < .001; * = p < .05; *italics:* not significant in the sensitivity analysis

**eTable 8: Mediation effect analyses and 95% bias-corrected bootstrap CI, sensitivity analysis in the community and the clinical sample**

|  | | **Model 1.2., community sample** (N=518) | | | | | | **Model 1.2., clinical sample** (N=327) | | |
| --- | --- | --- | --- | --- | --- | --- | --- | --- | --- | --- |
|  | | **Standardized coefficient** | | **p** | | | **95%CI** | **Standardized coefficient** | **p** | **95%CI** |
| **Mediation pathway** | | | | | | | | | | |
| ***Competence beliefs – adaptive coping – CHR symptoms*** | | | | | | | | | | |
| Indirect effect | |  |  | | |  | | *-0.028* | *0.112* | *-13.008, 0.830* |
| Total effect | |  |  | | |  | | -0.224 | 0.002* | 1.495, 72.304 |
| ***Adaptive LOC – adaptive coping – CHR symptoms*** | | | | | | | | | | |
| Indirect effect |  | |  | | |  | | *-0.027* | *0.104* | *-0.054,*  *2.560* |
| Total effect |  | |  | | |  | | *0.015* | *0.800* | *-12.467,*  *0.020* |
| ***Maladaptive LOC – maladaptive coping – PP*** | | | | | | | | | | |
| *Indirect effect* | | *0.208* | | *0.080* | | | *0.004, 0.038* |  |  |  |
| *Total effect* | | *0.169* | | *0.573* | | | *-0.009, 0.032* |  |  |  |
| ***Maladaptive LOC – maladaptive coping - SRH*** | | | | | | | | | | |
| *Indirect effect* | | *-0.213* | | | *0.080* | | *-0.715,*  *-0.084* |  |  |  |
| *Total effect* | | *-0.102* | | | *0.723* | | *-0.338, 0.492* |  |  |  |
| ***Maladaptive LOC – maladaptive coping – CHR symptoms*** | | | | | | | | | | |
| *Indirect effect* | | *0.076* | | | *0.516* | | *-0.003, 0.022* | *0.027* | *0.304* | *-0.007,*  *0.041* |
| *Total effect* | | *0.087* | | | *0.422* | | *-0.011, 0.024* | 0.155 | 0.008* | -2.246, 0.156 |
| ***Maladaptive coping – CHR symptoms – SRH*** | | | | | | | | | | |
| *Indirect effect* | | *-0.031* | | | *0.333* | | *-0.168, 0.011* | *-0.026* | *0.329* | *-6.838,*  *0.035* |
| Total effect | | -0.432 | | | 0.016* | | -1.472,  -0.256 | *-0.033* | *0.699* | *-0.774,*  *0.071* |
| ***Maladaptive coping – CHR symptoms – PP*** | | | | | | | | | | |
| *Indirect effect* | | *0.030* | | | *0.308* | | *-0.001, 0.007* | *0.019* | *0.326* | *-0.002,*  *0.369* |
| Total effect | | 0.422 | | | 0.007* | | 0.011, 0.052 | *-0.034* | *0.616* | -0.027, 0.004 |
| ***Adaptive coping – CHR symptoms – SRH*** | | | | | | | | | | |
| Indirect effect | |  | | |  | |  | *0.043* | *0.097* | *-19.236,*  *0.179* |
| Total effect | |  | | |  | |  | *0.046* | *0.564* | *-0.075,*  *5.894* |
| ***Adaptive coping – CHR symptoms – PP*** | | | | | | | | | | |
| Indirect effect | |  | | |  | |  | *-0.031* | *0.101* | *-0.012,*  *1.010* |
| Total effect | |  | | |  | |  | *-0.110* | *0.090* | *-1.536,*  *-0.003* |

*Note:* ** = p < .001; * = p < .05; *italics:* not significant; value missing: indirect effect was not analyzed in the corresponding sample.

**References Supplementary Material**

[1] Michel C, Schimmelmann BKE, Kupferschmid S, et al. Reliability of telephone assessments of at-risk criteria of psychosis: a comparison to face-to-face interviews. Schizophr Res 2014; 153: 251–3. https://doi.org/10.1016/j.schres.2014.01.025.

[2] Michel C, Schimmelmann BG, Schultze‐Lutter F. Demographic and clinical characteristics of diagnosed and non‐diagnosed psychotic disorders in the community. Early Interv Psychiatry 2018; 12: 87–90. https://doi.org/10.1111/eip.12360.

[3] Schultze-Lutter F, Michel C, Ruhrmann S, et al. Prevalence and clinical relevance of interview-assessed psychosis-risk symptoms in the young adult community. Psychol Med 2018; 48: 1167–78. https://doi.org/10.1017/S0033291717002586

[4] Schultze-Lutter F, Schimmelmann BG, Michel C. Clinical high-risk of and conversion to psychosis in the community: A 3-year follow-up of a cohort study. Schizophr Res 2021; 228: 616–8. https://doi.org/10.1016/j.schres.2020.11.032.

[5] Michel C, Kaess M, Flückiger R, et al. The Bern Early Recognition and Intervention Centre for mental crisis (FETZ Bern)—An 8‐year evaluation. Early Interv Psychiatry 2022; 16: 289–301. https://doi.org/10.1111/eip.13160.

[6] Schultze-Lutter F, Michel C, Schmidt SJ, et al. EPA guidance on the early detection of clinical high risk states of psychoses. Eur Psychiatry 2015; 30: 405–16. https://doi.org/10.1016/j.eurpsy.2015.01.010.

[7] Sheehan DV, Lecrubier Y, Sheehan KH, et al. The Mini-International Neuropsychiatric Interview (M.I.N.I): The development and validation of a structured diagnostic psychiatric interview for DSM-IV and ICD-10. J Clin Psychiatry 1998; 59: 22–33.

[8] American Psychiatric Association. Diagnostic and statistical manual of mental disorders. 4^th^ ed. Washington, DC: American Psychiatric Association; 1994.

[9] Hinz A, Klaiberg A, Brähler E, et al. The Quality of Life Questionnaire EQ-5D: modelling and norm values for the general population. Psychother Psychosom Med Psychol 2006; 56: 42–8. https://doi.org/ 10.1055/s-2005-867061.

[10] Michel C, Schmidt SJ, Schnyder N, et al. Associations of psychosis-risk symptoms with quality of life and self-rated health in the Community. Eur Psychiatry 2019; 62: 116–23. https://doi.org/10.1016/j.eurpsy.2019.08.008.

[11] Krampen G. Fragebogen zu Kompetenz-und Kontrollüberzeugungen (FKK): Handanweisung und Verbrauchsmaterialien. Göttingen: Hogrefe; 1991.

[12] Rotter JB. Generalized expectancies for internal versus external control of reinforcement. Psychol Monogr Gen Appl 1966; 80: 1–28. https://doi.org/10.1037/h0092976.

[13] Groth N, Schnyder N, Kaess M, et al. Coping as a mediator between locus of control, competence beliefs, and mental health: A systematic review and structural equation modelling meta-analysis. Behav Res Ther 2019; 121: 103442. https://doi.org/10.1016/j.brat.2019.103442.
